# *HTRA1* methylation in peripheral blood as a potential marker for the preclinical detection of stroke: a case–control study and a prospective nested case–control study

**DOI:** 10.1186/s13148-022-01418-0

**Published:** 2022-12-29

**Authors:** Chunlan Liu, Mengxia Li, Qiming Yin, Yao Fan, Chong Shen, Rongxi Yang

**Affiliations:** 1 Department of Epidemiology and Biostatistics, School of Public Health, Nanjing Medical University, 101 Longmian Avenue, Jiangning, Nanjing, 211166 China; 2 Division of Clinical Epidemiology, Affiliated Geriatric Hospital of Nanjing Medical University, Nanjing, 211166 China

**Keywords:** *HTRA1*, Methylation, Stroke, Preclinical detection, Marker

## Abstract

**Background:**

Stroke is the leading cause of mortality in China. DNA methylation has essential roles in multiple diseases, but its association with stroke was barely studied. We hereby explored the association between blood-based HTRA serine protease 1 (*HTRA1*) methylation and the risk of stroke.

**Results:**

The association was discovered in a hospital-based case–control study (cases/controls = 190:190) and further validated in a prospective nested case–control study including 139 cases who developed stroke within 2 years after recruitment and 144 matched stroke-free controls. We observed stroke-related altered *HTRA1* methylation and expression in both case–control study and prospective study. This blood-based *HTRA1* methylation was associated with stroke independently from the known risk factors and mostly affected the older population. The prospective results further showed that the altered *HTRA1* methylation was detectable 2 years before the clinical determination of stroke and became more robust with increased discriminatory power for stroke along with time when combined with other known stroke-related variables [onset time ≤ 1 year: area under the curve (AUC) = 0.76].

**Conclusions:**

In our study, altered *HTRA1* methylation was associated with stroke at clinical and preclinical stages and thus may provide a potential biomarker in the blood for the risk evaluation and preclinical detection of stroke.

**Supplementary Information:**

The online version contains supplementary material available at 10.1186/s13148-022-01418-0.

## Background

Stroke is a leading cause of mortality and a major contributor to neurological disability in low-income and middle-come countries [1, 2]. In 2019, stroke was responsible for 6.55 million deaths and 143.23 million disability-adjusted life years (DALYs) worldwide [2]. In China, there were 3.94 million new stroke cases and 2.19 million deaths with the highest DALYs of 45.9 million lost in 2019 [3].

Stroke is a complex disease attributed to genetic and environmental factors [4]. According to the genome-wide association studies, multiple genetic factors have only explained 5–10% of the risk for stroke [5, 6]. In recent years, attention has been paid to epigenetic factors and their role in the pathophysiological process of stroke [7]. DNA methylation is a crucial epigenetic modification involved in diabetes mellitus [8], atherosclerosis [9] and cardiovascular diseases [10]. DNA methylation can influence the structure of the genome and regulate the process of transcription, which mediates alterations in the expression of specific genes [11, 12]. Candidate approach studies have identified the association between stroke and altered blood-based DNA methylation in *LINE-1*, *ABCB1* and *CBS* genes, but mainly in case–control studies with small sample sizes [13–15]. Our previous study has also revealed that aberrant methylation of *ACTB* in peripheral blood was associated with the risk of stroke [16].

The HTRA serine protease 1 (encoded by *HTRA1* gene) is one of the human serine proteases that participate in diverse physiological processes including the maintenance of mitochondrial homeostasis, cell signaling and apoptosis [17]. Several studies have shown that heterozygous *HTRA1* mutations are closely related to cerebral small vessel disease (SVD), small-vessel ischemic stroke and lacunar stroke [18–23]. Our previous study also showed that *HTRA1* variants might confer the genetic susceptibility to stroke [24]. However, hereditary stroke attributable to *HTRA1* gene constitutes only a small proportion of all strokes. So far, no data are available about the association between blood-based *HTRA1* methylation and stroke, especially in the prospective studies.

In the present study, the association between peripheral blood-based *HTRA1* methylation and the risk of stroke in the Chinese population was evaluated in an independent case–control study consisting of 190 stroke patients and 190 age- and sex-matched controls and was further verified in a prospective nested case–control study including 139 cases who developed stroke within 2 years after recruitment and 144 matched stroke-free controls from a cohort with a total of 11,151 subjects.

## Results

### Demographic characteristics of the subjects

The demographic and clinical characteristics of subjects in the case–control study and prospective nested case–control study are presented in Table 1. In the case–control study, the stroke cases had a higher proportion of drinking, higher prevalence of hypertension and diabetes, and higher levels of diastolic blood pressure (DBP), but lower levels of high-density lipoprotein cholesterol (HDL-C) than controls (all *P* < 0.05). No significant difference existed in age, sex, smoking status, systolic blood pressure (SBP), total cholesterol (TC), triglycerides (TG), low-density lipoprotein cholesterol (LDL-C), and fasting plasma glucose between the two groups.

**Table 1:** Demographic and clinical characteristics of subjects

Characteristics	Group	Case–control study				Prospective nested case–control study			
		Controls	Stroke cases	*t*/*χ*^2^	*P* value	Controls	Stroke cases	*t*/*χ*^2^	*P* value
		(*n* = 190)	(*n* = 190)			(*n* = 144)	(*n* = 139)		
Age (mean ± SD, years)		65.89 ± 10.12	64.99 ± 9.85	0.884	0.377	67.77 ± 9.11	67.64 ± 9.51	0.117	0.907
Sex	Male	83 (43.7%)	79 (41.6%)	0.172	0.678	83 (57.6%)	81 (58.3%)	0.012	0.914
	Female	107 (56.3%)	111 (58.4%)			61 (42.4%)	58 (41.7%)		
SBP (mean ± SD, mmHg)		148.65 ± 19.76	152.44 ± 22.27	1.748	0.081	147.74 ± 20.19	147.73 ± 22.28	0.005	0.996
DBP (mean ± SD, mmHg)		80.46 ± 11.75	85.62 ± 14.42	3.814	1.60E−04	81.50 ± 9.75	81.16 ± 11.86	0.262	0.794
Smoking (*n*, %)	Yes	53 (28.0%)	47 (24.7%)	0.533	0.465	36 (25.0%)	35 (25.2%)	0.001	0.972
	No	136 (72.0%)	143 (75.3%)			108 (75.0%)	104 (74.8%)		
Drinking (*n*, %)	Yes	69 (36.5%)	30 (15.8%)	21.075	4.00E−06	47 (32.6%)	47 (33.8%)	0.044	0.834
	No	120 (63.5%)	160 (84.2%)			97 (67.4%)	92 (66.2%)		
History of hypertension (*n*, %)	Yes	155 (82.0%)	125 (65.8%)	12.919	0.003	87 (60.4%)	96 (69.1%)	2.315	0.128
	No	34 (18.0%)	65 (34.2%)			57 (39.6%)	43 (30.9%)		
History of diabetes (*n*, %)	Yes	71 (37.6%)	51 (26.8%)	4.992	0.025	34 (23.6%)	42 (30.2%)	1.571	0.210
	No	118 (62.4%)	139 (73.2%)			110 (76.4%)	97 (69.8%)		
TC (mean ± SD, mmol/L)		4.84 ± 0.82	4.97 ± 4.42	0.392	0.695	5.11 ± 0.91	5.20 ± 0.93	0.869	0.386
TG (mean ± SD, mmol/L)		1.64 ± 1.29	1.80 ± 1.45	1.113	0.267	1.56 ± 1.00	1.56 ± 0.88	0.014	0.989
HDL-C (mean ± SD, mmol/L)		1.49 ± 0.39	1.27 ± 0.38	5.453	9.13E−08	1.54 ± 0.46	1.55 ± 0.42	0.179	0.858
LDL-C (mean ± SD, mmol/L)		2.65 ± 0.69	2.76 ± 2.00	0.744	0.457	2.88 ± 0.80	2.96 ± 0.75	0.848	0.397
Fasting glucose (mean ± SD, mmol/L)		6.77 ± 2.01	6.62 ± 7.26	0.254	0.800	6.61 ± 2.26	6.64 ± 2.24	0.126	0.900

*SBP* systolic blood pressure, *DBP* diastolic blood pressure, *TC* total cholesterol, *TG* triglycerides, *HDL-C* high-density lipoprotein cholesterol, *LDL-C* low-density lipoprotein cholesterol

In the prospective nested case–control study, there were no significant differences in age, sex, SBP, DBP, smoking status, drinking status, history of hypertension, diabetes, levels of TC, TG, HDL-C, LDL-C and fasting plasma glucose between stroke cases and controls. The median time between blood draw and initial diagnosis of stroke in cases was 1.32 years.

### Association between *HTRA1* methylation and stroke in the case–control study and the prospective nested case–control study

To evaluate the association between *HTRA1* methylation and the risk of stroke in the case–control study and the prospective nested case–control study, two amplicons, HTRA1-A and HTRA1-B, including 17 measurable CpG sites were semiquantitatively determined by Agena MALDI-TOF mass spectrometry.

In the case–control study (190 cases vs. 190 controls), HTRA1_A_CpG_6 showed significantly lower methylation levels in stroke cases than in the controls [median = 0.10 (interquartile range, IQR = 0.06–0.13) and 0.12 (IQR = 0.10–0.15) for cases and controls, respectively, OR per + 10% methylation = 0.54, 95% CI 0.34–0.86, *P* = 0.009 by logistic regression adjusted for age, sex, smoking, drinking, hypertension, diabetes, TC, TG, HDL-C and LDL-C, Table 2]. The other CpG sites in the HTRA1 amplicons showed no significant associations with stroke (Table 2).

**Table 2:** Methylation difference of *HTRA1* between 190 stroke cases and 190 controls in the case–control study

CpG sites	Controls	Stroke cases	Crude OR (95% CI)	*P* value	OR (95% CI)^☆^	*P* value^☆^	OR (95% CI)*	*P* value*
	Median (IQR)	Median (IQR)	Per + 10% methylation		Per + 10% methylation		Per + 10% methylation	
HTRA1_A_CpG_1	0.09 (0.05–0.12)	0.10 (0.07–0.13)	1.27 (0.92–1.75)	0.146	1.27 (0.92–1.76)	0.140	1.22 (0.85–1.77)	0.284
HTRA1_A_CpG_2	0.65 (0.56–0.73)	0.64 (0.58–0.74)	1.01 (0.92–1.12)	0.803	1.01 (0.92–1.12)	0.821	0.99 (0.88–1.11)	0.860
HTRA1_A_CpG_3	0.29 (0.22–0.35)	0.29 (0.24–0.35)	0.96 (0.78–1.17)	0.662	0.95 (0.78–1.16)	0.636	0.92 (0.73–1.15)	0.466
HTRA1_A_CpG_4	0.12 (0.09–0.15)	0.13 (0.11–0.15)	1.48 (0.96–2.30)	0.078	1.47 (0.95–2.28)	0.087	1.29 (0.79–2.11)	0.310
HTRA1_A_CpG_5	0.37 (0.31–0.45)	0.38 (0.30–0.45)	0.99 (0.86–1.14)	0.891	0.99 (0.86–1.14)	0.903	0.99 (0.85–1.16)	0.913
HTRA1_A_CpG_6	0.12 (0.10–0.15)	0.10 (0.06–0.13)	0.50 (0.32–0.78)	0.002	0.51 (0.33–0.79)	0.003	0.54 (0.34–0.86)	**0.009**
HTRA1_A_CpG_7	0.36 (0.28–0.45)	0.37 (0.30–0.45)	1.08 (0.92–1.27)	0.343	1.07 (0.92–1.26)	0.378	1.09 (0.91–1.30)	0.369
HTRA1_B_CpG_1	0.16 (0.09–0.41)	0.18 (0.11–0.44)	1.04 (0.95–1.13)	0.384	1.04 (0.96–1.14)	0.333	1.05 (0.95–1.16)	0.308
HTRA1_B_CpG_2	0.09 (0.05–0.12)	0.10 (0.07–0.14)	1.45 (1.02–2.06)	0.039	1.45 (1.02–2.06)	0.040	1.25 (0.86–1.82)	0.243
HTRA1_B_CpG_3.4	0.28 (0.24–0.33)	0.29 (0.25–0.34)	1.18 (0.90–1.55)	0.236	1.19 (0.90–1.57)	0.221	1.11 (0.81–1.52)	0.513
HTRA1_B_CpG_5	0.26 (0.20–0.34)	0.28 (0.22–0.33)	1.09 (0.90–1.33)	0.378	1.10 (0.90–1.34)	0.367	1.06 (0.85–1.32)	0.612
HTRA1_B_CpG_7	0.47 (0.36–0.54)	0.47 (0.37–0.54)	1.06 (0.92–1.22)	0.456	1.06 (0.92–1.22)	0.448	1.01 (0.85–1.19)	0.957
HTRA1_B_CpG_8.9	0.28 (0.24–0.33)	0.29 (0.25–0.34)	1.18 (0.90–1.55)	0.236	1.19 (0.90–1.57)	0.221	1.11 (0.81–1.52)	0.513
HTRA1_B_CpG_10	0.61 (0.53–0.71)	0.63 (0.55–0.70)	1.06 (0.92–1.23)	0.445	1.06 (0.92–1.23)	0.423	1.03 (0.87–1.22)	0.722
HTRA1_B_CpG_11.12	0.72 (0.65–0.80)	0.73 (0.66–0.80)	1.09 (0.92–1.30)	0.315	1.10 (0.92–1.31)	0.300	1.07 (0.88–1.31)	0.504
HTRA1_B_CpG_13.14.15	0.49 (0.41–0.57)	0.51 (0.45–0.57)	1.15 (0.97–1.36)	0.115	1.15 (0.97–1.36)	0.109	1.12 (0.92–1.36)	0.261
HTRA1_B_CpG_16	0.53 (0.45–0.63)	0.53 (0.45–0.68)	1.03 (0.91–1.17)	0.636	1.03 (0.91–1.17)	0.658	0.99 (0.86–1.15)	0.911

Bold values indicated *P* < 0.05

^☆^Logistic regression, adjusted for age and sex;

^*^Logistic regression, adjusted for age, sex, smoking, drinking, hypertension, diabetes, TC, TG, HDL-C and LDL-C

**Fig. 1:**
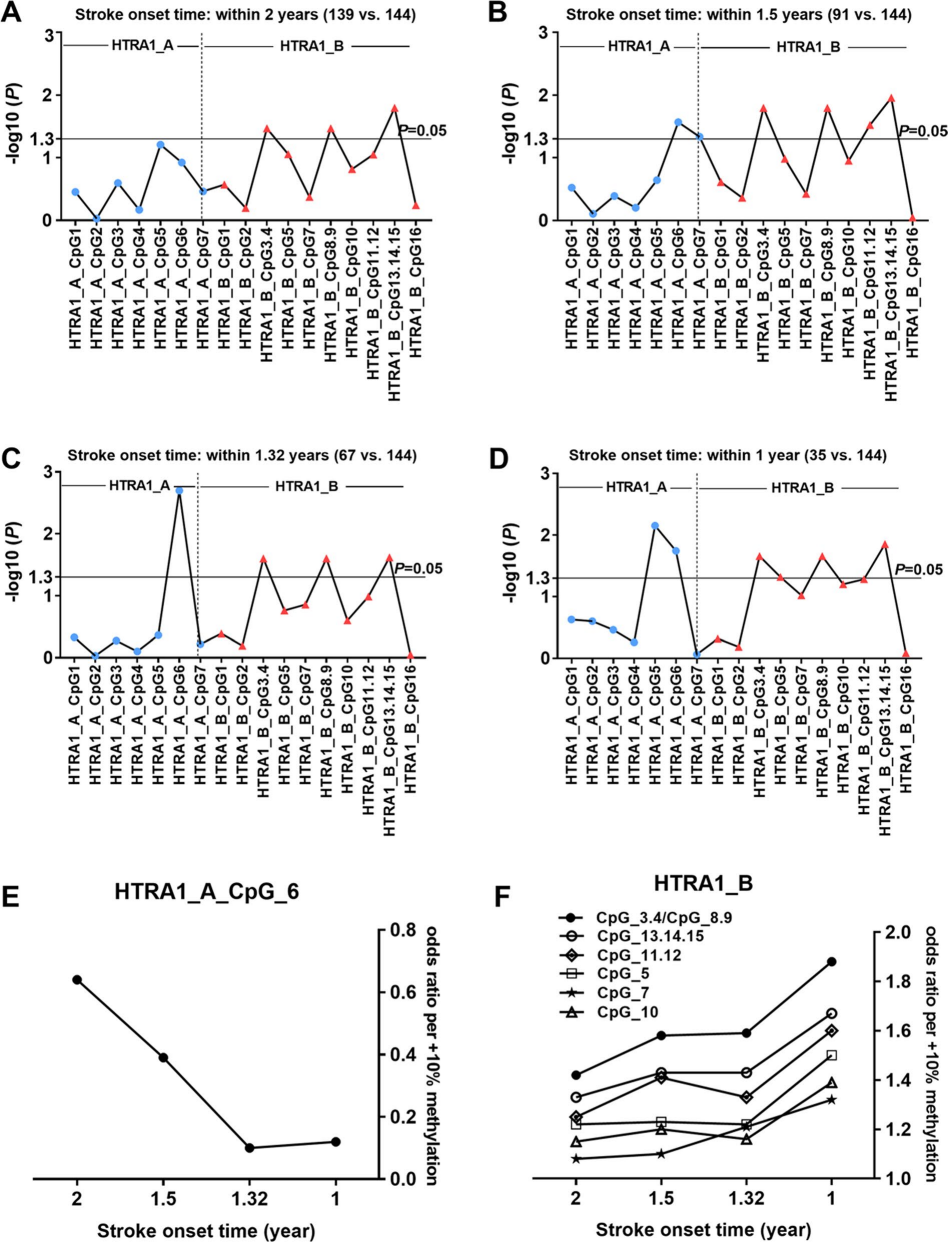
Onset-time-dependent association between *HTRA1* methylation and stroke in the prospective nested case–control study. The analysis was performed for stroke cases with onset time **A** < 2 years, **B** ≤ 1.5 years, **C** ≤ 1.32 years, and **D** ≤ 1 year. The *P* values of all the 17 measureable CpG loci in the HTRA1-A and HTRA1-B amplicons were calculated by logistic regression adjusting for age, sex, smoking, drinking, hypertension, diabetes, TC, TG, HDL-C and LDL-C, and all the *P* values were transformed by -log_10._ The dotted lines indicate the thresholds of different *P* values of 0.05. **E** and **F** The OR per + 10% methylation of HTRA1_A_CpG_6 and CpG_3.4, CpG_5, CpG_7, CpG_8.9, CpG_10, CpG_11.12 as well as CpG_13.14.15 in the HTRA1-B amplicon in stroke cases with onset time < 2 years, ≤ 1.5 years, ≤ 1.32 years and ≤ 1 year based on logistic regression analysis adjusting for age, sex, smoking, drinking, hypertension, diabetes, TC, TG, HDL-C and LDL-C

In the prospective nested case–control study, comparing 144 controls and all 139 cases who developed stroke within 2 years, the methylation levels of three measurable CpG loci (CpG_3.4, CpG_8.9, and CpG_13.14.15) in the HTRA1-B amplicon were significantly higher in cases than in controls (ORs per + 10% methylation from 1.33 to 1.42, *P* ≤ 0.034 for all by logistic regression adjusted for age, sex, smoking, drinking, hypertension, diabetes, TC, TG, HDL-C and LDL-C, Fig. 1A, Additional file 1: Table S1).

Since the onset time of stroke may have an effect on the methylation levels of *HTRA1*, we further performed analyses comparing controls and stroke cases stratified by time of stroke onset in the prospective nested case–control study. Interestingly, the differences between the aforementioned three CpG loci became more pronounced in stroke cases with onset time ≤ 1.5 years (ORs per + 10% methylation from 1.43 to 1.58, *P* ≤ 0.016 for all by logistic regression adjusted for age, sex, smoking, drinking, hypertension, diabetes, TC, TG, HDL-C and LDL-C, Fig. 1B, Additional file 1: Table S2) and in cases with onset time ≤ 1.32 years (ORs per + 10% methylation from 1.43 to 1.59, *P* ≤ 0.025 for all by logistic regression adjusted for age, sex, smoking, drinking, hypertension, diabetes, TC, TG, HDL-C and LDL-C, Fig. 1C, Additional file 1: Table S3), especially in stroke cases with onset time ≤ 1 year (ORs per + 10% methylation from 1.67 to 1.88, *P* ≤ 0.022 for all by logistic regression adjusted for age, sex, smoking, drinking, hypertension, diabetes, TC, TG, HDL-C and LDL-C, Fig. 1D, Additional file 1: Table S4). Additionally, CpG_5, CpG_7, CpG_10 and CpG_11.12 in the HTRA1-B amplicon showed borderline associations with stroke in cases with shorter onset time (Fig. 1A–D, Additional file 1: Tables S1–S4). Moreover, stroke cases with onset time ≤ 1.5 years showed significantly lower HTRA1_A_CpG_6 methylation levels than controls (OR per + 10% methylation = 0.39, 95% CI 0.17–0.90, *P* = 0.027 by logistic regression adjusted for age, sex, smoking, drinking, hypertension, diabetes, TC, TG, HDL-C and LDL-C, Fig. 1B, Additional file 1: Table S2). Similarly, the differences became more pronounced in stroke cases with onset time ≤ 1.32 years (OR per + 10% methylation = 0.10, 95% CI 0.03–0.42, *P* = 0.002 by logistic regression adjusted for age, sex, smoking, drinking, hypertension, diabetes, TC, TG, HDL-C and LDL-C, Fig. 1C, Additional file 1: Table S3), and in cases with onset time ≤ 1 year (OR per + 10% methylation = 0.12, 95% CI 0.02–0.70, *P* = 0.018 by logistic regression adjusted for age, sex, smoking, drinking, hypertension, diabetes, TC, TG, HDL-C and LDL-C, Fig. 1D, Additional file 1: Table S4). HTRA1_A_CpG_7 and HTRA1_A_CpG_5 also showed significant association with stroke in cases with onset time ≤ 1.5 years and ≤ 1 year, respectively (*P* = 0.046 and 0.007 by logistic regression adjusted for age, sex, smoking, drinking, hypertension, diabetes, TC, TG, HDL-C and LDL-C, Fig. 1B and D Additional file 1: Table S2, Additional file 1: Table S4). Overall, the OR per + 10% methylation showed clear trends of decrease in HTRA1_A_CpG_6 in stroke cases with onset time < 2 years, ≤ 1.5 years, ≤ 1.32 years and ≤ 1 year (Fig. 1E). The OR per + 10% methylation showed clear trends of increase in CpG_3.4, CpG_5, CpG_7, CpG_8.9, CpG_10, CpG_11.12 and CpG_13.14.15 in the HTRA1-B amplicon in stroke cases with onset time < 2 years, ≤ 1.5 years, ≤ 1.32 years and ≤ 1 year (Fig. 1F).

### Association between blood-based methylation of *HTRA1* and stroke in the older population

Age was reported to play an essential role in the pattern of DNA methylation [25]; we therefore evaluated the association between *HTRA1* methylation in the blood and stroke stratified by two major age groups (< 70 and ≥ 70 years). In the case–control study, stroke cases showed significantly lower methylation levels of HTRA1_A_CpG_3 and HTRA1_A_CpG_6 than controls among participants ≥ 70 years old (ORs per + 10% methylation = 0.63 and 0.29, *P* = 0.022 and 0.008, respectively, by logistic regression adjusted for age, sex, smoking, drinking, hypertension, diabetes, TC, TG, HDL-C and LDL-C, Table 3). However, no association between *HTRA1* methylation and stroke was observed among subjects < 70 years old (Table 3).

**Table 3:** Methylation difference of *HTRA1* between stroke cases and controls stratified by age in the case–control study

CpG sites	Controls	Stroke cases	Crude OR (95% CI)	*P* value	OR (95% CI)^☆^	*P* value^☆^	OR (95% CI)*	*P* value*
	Median (IQR)	Median (IQR)	Per + 10% methylation		Per + 10% methylation		Per + 10% methylation	
*Participants* < *70 years old (116 stroke cases vs. 125 controls)*								
HTRA1_A_CpG_1	0.09 (0.05–0.13)	0.11 (0.08–0.13)	1.23 (0.84–1.81)	0.293	1.23 (0.84–1.81)	0.293	1.20 (0.76–1.89)	0.448
HTRA1_A_CpG_2	0.65 (0.57–0.79)	0.64 (0.56–0.74)	0.98 (0.86–1.11)	0.707	0.98 (0.86–1.11)	0.696	0.96 (0.83–1.10)	0.510
HTRA1_A_CpG_3	0.28 (0.22–0.35)	0.30 (0.25–0.35)	1.10 (0.85–1.41)	0.484	1.10 (0.85–1.41)	0.489	1.13 (0.85–1.51)	0.409
HTRA1_A_CpG_4	0.12 (0.09–0.15)	0.13 (0.11–0.15)	1.31 (0.74–2.30)	0.354	1.30 (0.74–2.29)	0.369	1.02 (0.54–1.94)	0.951
HTRA1_A_CpG_5	0.37 (0.31–0.43)	0.38 (0.30–0.43)	0.96 (0.81–1.15)	0.679	0.96 (0.81–1.15)	0.680	0.97 (0.79–1.18)	0.731
HTRA1_A_CpG_6	0.12 (0.10–0.14)	0.10 (0.06–0.14)	0.66 (0.40–1.08)	0.097	0.66 (0.40–1.09)	0.106	0.69 (0.41–1.16)	0.163
HTRA1_A_CpG_7	0.36 (0.28–0.45)	0.37 (0.31–0.45)	1.04 (0.86–1.27)	0.685	1.04 (0.85–1.27)	0.713	1.06 (0.85–1.34)	0.602
HTRA1_B_CpG_1	0.16 (0.09–0.40)	0.18 (0.11–0.50)	1.09 (0.98–1.21)	0.122	1.09 (0.98–1.22)	0.123	1.11 (0.98–1.25)	0.107
HTRA1_B_CpG_2	0.09 (0.05–0.12)	0.11 (0.07–0.15)	1.66 (0.93–3.03)	0.102	1.65 (0.92–3.01)	0.103	1.80 (0.96–3.23)	0.062
HTRA1_B_CpG_3.4	0.28 (0.23–0.32)	0.29 (0.25–0.34)	1.41 (0.97–2.03)	0.069	1.42 (0.98–2.05)	0.063	1.29 (0.85–1.96)	0.230
HTRA1_B_CpG_5	0.25 (0.19–0.33)	0.28 (0.22–0.33)	1.19 (0.92–1.54)	0.190	1.20 (0.93–1.56)	0.169	1.18 (0.88–1.58)	0.274
HTRA1_B_CpG_7	0.47 (0.35–0.54)	0.46 (0.38–0.53)	1.07 (0.88–1.29)	0.520	1.06 (0.88–1.29)	0.535	0.99 (0.79–1.23)	0.906
HTRA1_B_CpG_8.9	0.28 (0.23–0.32)	0.29 (0.25–0.34)	1.41 (0.97–2.03)	0.069	1.42 (0.98–2.05)	0.063	1.29 (0.85–1.96)	0.230
HTRA1_B_CpG_10	0.61 (0.53–0.71)	0.64 (0.55–0.70)	1.14 (0.94–1.38)	0.180	1.14 (0.94–1.38)	0.173	1.13 (0.92–1.40)	0.249
HTRA1_B_CpG_11.12	0.72 (0.64–0.79)	0.73 (0.67–0.80)	1.13 (0.89–1.43)	0.330	1.12 (0.89–1.42)	0.343	1.11 (0.85–1.45)	0.451
HTRA1_B_CpG_13.14.15	0.48 (0.40–0.56)	0.50 (0.45–0.57)	1.24 (0.99–1.55)	0.066	1.24 (0.99–1.55)	0.068	1.19 (0.92–1.55)	0.186
HTRA1_B_CpG_16	0.51 (0.43–0.62)	0.53 (0.44–0.66)	1.08 (0.92–1.27)	0.367	1.08 (0.92–1.28)	0.356	1.03 (0.85–1.24)	0.800
*Participants* ≥ *70 years old (74 stroke cases vs. 65 controls)*								
HTRA1_A_CpG_1	0.09 (0.06–0.12)	0.10 (0.07–0.14)	1.38 (0.77–2.45)	0.278	1.37 (0.77–2.44)	0.282	1.43 (0.73–2.82)	0.300
HTRA1_A_CpG_2	0.63 (0.56–0.71)	0.65 (0.59–0.78)	1.08 (0.92–1.27)	0.335	1.09 (0.92–1.28)	0.340	1.05 (0.85–1.30)	0.631
HTRA1_A_CpG_3	0.32 (0.25–0.38)	0.28 (0.22–0.36)	0.76 (0.54–1.05)	0.097	0.74 (0.53–1.04)	0.087	0.63 (0.42–0.94)	**0.022**
HTRA1_A_CpG_4	0.12 (0.09–0.15)	0.12 (0.11–0.15)	1.79 (0.87–3.71)	0.116	1.80 (0.87–3.74)	0.113	1.92 (0.78–4.70)	0.156
HTRA1_A_CpG_5	0.37 (0.3–0.46)	0.38 (0.29–0.49)	1.04 (0.82–1.32)	0.750	1.04 (0.82–1.32)	0.752	1.01 (0.76–1.33)	0.967
HTRA1_A_CpG_6	0.12 (0.10–0.15)	0.10 (0.07–0.13)	0.27 (0.12–0.63)	0.003	0.27 (0.11–0.62)	0.002	0.29 (0.12–0.73)	**0.008**
HTRA1_A_CpG_7	0.36 (0.27–0.46)	0.38 (0.30–0.48)	1.16 (0.88–1.53)	0.282	1.15 (0.88–1.52)	0.310	1.12 (0.81–1.53)	0.497
HTRA1_B_CpG_1	0.15 (0.09–0.42)	0.17 (0.09–0.42)	0.96 (0.83–1.10)	0.557	0.95 (0.83–1.10)	0.529	0.94 (0.79–1.13)	0.511
HTRA1_B_CpG_2	0.08 (0.05–0.14)	0.09 (0.07–0.12)	0.92 (0.56–1.52)	0.756	0.92 (0.56–1.52)	0.749	0.70 (0.39–1.28)	0.251
HTRA1_B_CpG_3.4	0.30 (0.25–0.34)	0.29 (0.23–0.34)	0.92 (0.60–1.41)	0.701	0.93 (0.60–1.44)	0.737	0.85 (0.50–1.43)	0.534
HTRA1_B_CpG_5	0.27 (0.22–0.36)	0.28 (0.21–0.34)	0.96 (0.71–1.30)	0.779	0.97 (0.71–1.32)	0.822	0.87 (0.60–1.26)	0.455
HTRA1_B_CpG_7	0.48 (0.38–0.55)	0.49 (0.34–0.56)	1.04 (0.84–1.29)	0.731	1.05 (0.84–1.32)	0.650	1.00 (0.75–1.33)	0.999
HTRA1_B_CpG_8.9	0.30 (0.25–0.34)	0.29 (0.23–0.34)	0.92 (0.60–1.41)	0.701	0.93 (0.60–1.44)	0.737	0.85 (0.50–1.43)	0.534
HTRA1_B_CpG_10	0.63 (0.55–0.73)	0.62 (0.53–0.72)	0.94 (0.74–1.19)	0.615	0.94 (0.74–1.20)	0.619	0.78 (0.57–1.07)	0.117
HTRA1_B_CpG_11.12	0.72 (0.66–0.82)	0.72 (0.65–0.82)	1.06 (0.81–1.37)	0.685	1.06 (0.81–1.39)	0.669	0.96 (0.67–1.37)	0.815
HTRA1_B_CpG_13.14.15	0.51 (0.42–0.62)	0.51 (0.44–0.57)	1.02 (0.79–1.32)	0.863	1.03 (0.79–1.34)	0.819	0.98 (0.70–1.38)	0.914
HTRA1_B_CpG_16	0.55 (0.47–0.67)	0.53 (0.45–0.69)	0.95 (0.78–1.17)	0.642	0.96 (0.78–1.17)	0.659	0.92 (0.72–1.17)	0.496

Bold values indicated *P* < 0.05

^☆^logistic regression, adjusted for age and sex

^*^logistic regression, adjusted for age, sex, smoking, drinking, hypertension, diabetes, TC, TG, HDL-C and LDL-C

In the prospective nested case–control study, the methylation levels of CpG_3.4, CpG_5, CpG_8.9 and CpG_13.14.15 in the HTRA1_B amplicon were significantly higher in stroke cases than in controls among participants ≥ 70 years old (ORs per + 10% methylation from 1.70 to 2.36, *P* ≤ 0.021 for all by logistic regression adjusting for age, sex, smoking, drinking, hypertension, diabetes, TC, TG, HDL-C and LDL-C, Table 4). This association was not observed in subjects < 70 years old in the prospective nested case–control study as well (Table 4).

**Table 4:** Methylation difference of *HTRA1* between stroke cases and controls stratified by age in the prospective nested case–control study

CpG sites	Controls	Stroke cases	Crude OR (95% CI)	*P* value	OR (95% CI)^☆^	*P* value^☆^	OR (95% CI)*	*P* value*
	Median (IQR)	Median (IQR)	Per + 10% methylation		Per + 10% methylation		Per + 10% methylation	
*Participants* < *70 years old (88 stroke cases vs. 90 controls)*								
HTRA1_A_CpG_1	0.07 (0.05–0.10)	0.07 (0.05–0.09)	0.72 (0.33–1.61)	0.427	0.72 (0.33–1.61)	0.428	0.67 (0.30–1.54)	0.348
HTRA1_A_CpG_2	0.78 (0.74–0.84)	0.77 (0.73–0.82)	1.09 (0.85–1.40)	0.504	1.08 (0.84–1.39)	0.542	1.15 (0.88–1.50)	0.316
HTRA1_A_CpG_3	0.44 (0.41–0.51)	0.46 (0.41–0.52)	1.02 (0.75–1.39)	0.899	1.05 (0.76–1.44)	0.772	1.05 (0.75–1.46)	0.780
HTRA1_A_CpG_4	0.19 (0.16–0.21)	0.20 (0.17–0.22)	1.39 (0.65–2.99)	0.400	1.38 (0.63–2.99)	0.421	1.54 (0.69–3.43)	0.295
HTRA1_A_CpG_5	0.41 (0.35–0.49)	0.41 (0.35–0.46)	0.89 (0.67–1.17)	0.394	0.88 (0.66–1.16)	0.365	0.87 (0.65–1.16)	0.339
HTRA1_A_CpG_6	0.09 (0.08–0.11)	0.08 (0.07–0.10)	0.59 (0.30–1.18)	0.135	0.57 (0.28–1.14)	0.110	0.55 (0.27–1.12)	0.101
HTRA1_A_CpG_7	0.39 (0.34–0.45)	0.40 (0.35–0.45)	1.20 (0.82–1.76)	0.343	1.23 (0.84–1.81)	0.297	1.22 (0.82–1.81)	0.320
HTRA1_B_CpG_1	0.19 (0.11–0.29)	0.18 (0.11–0.30)	1.11 (0.91–1.36)	0.299	1.11 (0.91–1.36)	0.322	1.14 (0.93–1.41)	0.208
HTRA1_B_CpG_2	0.12 (0.09–0.16)	0.13 (0.08–0.17)	1.17 (0.69–2.01)	0.560	1.19 (0.69–2.04)	0.534	1.19 (0.69–2.07)	0.528
HTRA1_B_CpG_3.4	0.31 (0.26–0.35)	0.33 (0.27–0.37)	1.23 (0.85–1.79)	0.278	1.21 (0.83–1.77)	0.321	1.22 (0.83–1.79)	0.312
HTRA1_B_CpG_5	0.34 (0.28–0.40)	0.34 (0.26–0.43)	1.06 (0.80–1.41)	0.688	1.05 (0.79–1.39)	0.751	1.04 (0.78–1.40)	0.781
HTRA1_B_CpG_7	0.47 (0.39–0.53)	0.46 (0.38–0.58)	1.04 (0.83–1.32)	0.720	1.03 (0.81–1.30)	0.826	1.03 (0.80–1.31)	0.833
HTRA1_B_CpG_8.9	0.31 (0.26–0.35)	0.33 (0.27–0.37)	1.23 (0.85–1.79)	0.278	1.21 (0.83–1.77)	0.321	1.22 (0.83–1.79)	0.312
HTRA1_B_CpG_10	0.61 (0.55–0.69)	0.66 (0.56–0.75)	1.17 (0.91–1.51)	0.233	1.17 (0.90–1.51)	0.236	1.18 (0.90–1.54)	0.224
HTRA1_B_CpG_11.12	0.75 (0.69–0.79)	0.75 (0.70–0.82)	1.14 (0.83–1.58)	0.428	1.12 (0.81–1.55)	0.509	1.11 (0.79–1.55)	0.558
HTRA1_B_CpG_13.14.15	0.54 (0.46–0.61)	0.56 (0.47–0.61)	1.17 (0.88–1.54)	0.281	1.15 (0.87–1.53)	0.314	1.16 (0.87–1.54)	0.317
HTRA1_B_CpG_16	0.48 (0.42–0.58)	0.49 (0.37–0.57)	0.92 (0.76–1.12)	0.393	0.91 (0.75–1.11)	0.351	0.90 (0.74–1.10)	0.321
*Participants* ≥ *70 years old (51 stroke cases vs. 53 controls)*								
HTRA1_A_CpG_1	0.07 (0.06–0.08)	0.07 (0.05–0.09)	0.73 (0.25–2.13)	0.563	0.66 (0.22–1.98)	0.457	0.67 (0.20–2.22)	0.511
HTRA1_A_CpG_2	0.76 (0.71–0.82)	0.76 (0.71–0.81)	0.86 (0.65–1.14)	0.303	0.87 (0.65–1.16)	0.330	0.86 (0.60–1.23)	0.418
HTRA1_A_CpG_3	0.44 (0.38–0.47)	0.46 (0.42–0.54)	1.78 (1.08–2.93)	0.024	1.77 (1.06–2.93)	0.028	1.69 (0.97–2.93)	0.063
HTRA1_A_CpG_4	0.20 (0.17–0.22)	0.19 (0.17–0.21)	1.02 (0.50–2.09)	0.962	1.01 (0.49–2.10)	0.975	0.90 (0.40–2.02)	0.803
HTRA1_A_CpG_5	0.44 (0.38–0.51)	0.40 (0.34–0.45)	0.71 (0.50–1.01)	0.054	0.72 (0.50–1.02)	0.065	0.62 (0.40–1.01)	0.053
HTRA1_A_CpG_6	0.09 (0.08–0.10)	0.08 (0.07–0.10)	0.94 (0.35–2.48)	0.895	1.02 (0.38–2.74)	0.973	0.99 (0.33–2.96)	0.988
HTRA1_A_CpG_7	0.39 (0.35–0.42)	0.40 (0.34–0.48)	1.10 (0.69–1.75)	0.688	1.06 (0.66–1.71)	0.806	0.91 (0.54–1.52)	0.711
HTRA1_B_CpG_1	0.17 (0.12–0.25)	0.18 (0.10–0.29)	1.08 (0.85–1.36)	0.530	1.06 (0.84–1.35)	0.621	0.98 (0.75–1.28)	0.900
HTRA1_B_CpG_2	0.13 (0.09–0.17)	0.11 (0.08–0.16)	0.94 (0.52–1.69)	0.832	0.98 (0.54–1.77)	0.936	1.18 (0.60–2.31)	0.636
HTRA1_B_CpG_3.4	0.30 (0.25–0.35)	0.32 (0.27–0.37)	1.72 (0.97–3.06)	0.064	1.73 (0.97–3.08)	0.064	2.36 (1.18–4.73)	**0.015**
HTRA1_B_CpG_5	0.31 (0.26–0.39)	0.38 (0.28–0.47)	1.50 (1.03–2.19)	0.033	1.59 (1.07–2.37)	0.023	1.70 (1.08–2.67)	**0.021**
HTRA1_B_CpG_7	0.49 (0.40–0.53)	0.48 (0.41–0.57)	1.10 (0.82–1.48)	0.540	1.09 (0.81–1.47)	0.583	1.09 (0.78–1.52)	0.614
HTRA1_B_CpG_8.9	0.30 (0.25–0.35)	0.32 (0.27–0.37)	1.72 (0.97–3.06)	0.064	1.73 (0.97–3.08)	0.064	2.36 (1.18–4.73)	**0.015**
HTRA1_B_CpG_10	0.64 (0.54–0.69)	0.68 (0.53–0.76)	1.14 (0.87–1.49)	0.333	1.14 (0.87–1.49)	0.338	1.23 (0.91–1.65)	0.178
HTRA1_B_CpG_11.12	0.73 (0.68–0.78)	0.77 (0.68–0.83)	1.54 (1.01–2.35)	0.043	1.49 (0.98–2.28)	0.064	1.35 (0.85–2.15)	0.203
HTRA1_B_CpG_13.14.15	0.53 (0.47–0.57)	0.57 (0.50–0.67)	1.76 (1.16–2.69)	0.008	1.78 (1.15–2.76)	0.009	1.76 (1.09–2.86)	**0.021**
HTRA1_B_CpG_16	0.47 (0.39–0.55)	0.49 (0.37–0.60)	1.02 (0.79–1.30)	0.901	1.03 (0.80–1.32)	0.837	1.04 (0.79–1.37)	0.763

Bold values indicated *P* < 0.05

^☆^logistic regression, adjusted for age and sex

^*^logistic regression, adjusted for age, sex, smoking, drinking, hypertension, diabetes, TC, TG, HDL-C and LDL-C

### Correlation between *HTRA1* methylation and stroke-related characteristics

To explore the relationship between the blood-based *HTRA1* methylation and stroke-related characteristics, the subjects including both stroke cases and controls with available data were interpreted. In the case–control study, weak correlations were observed between the methylation levels of a few CpG sites in the HTRA1 amplicons and hypertension, HDL-C, current smoking status, TC as well as TG levels (Additional file 1: Table S5). None of the 17 CpG loci showed any correlation with drinking, diabetes and levels of LDL-C (Additional file 1: Table S5).

In the prospective nested case–control study, the methylation level of HTRA1_A_CpG_2 was positively correlated with current drinking status (Spearman rho = 0.199, *P* = 0.001, Additional file 1: Table S6), while the methylation levels of CpG_3, CpG_4 and CpG_5 in HTRA1_A amplicon were inversely correlated with hypertension (Spearman rho = − 0.121, − 0.263 and − 0.198, respectively, Additional file 1: Table S6). No correlations were observed between individual CpG sites and smoking, diabetes, levels of TC, TG, HDL-C and LDL-C (Additional file 1: Table S6).

### Expression of *HTRA1* in peripheral blood leukocytes

**Fig. 2:**
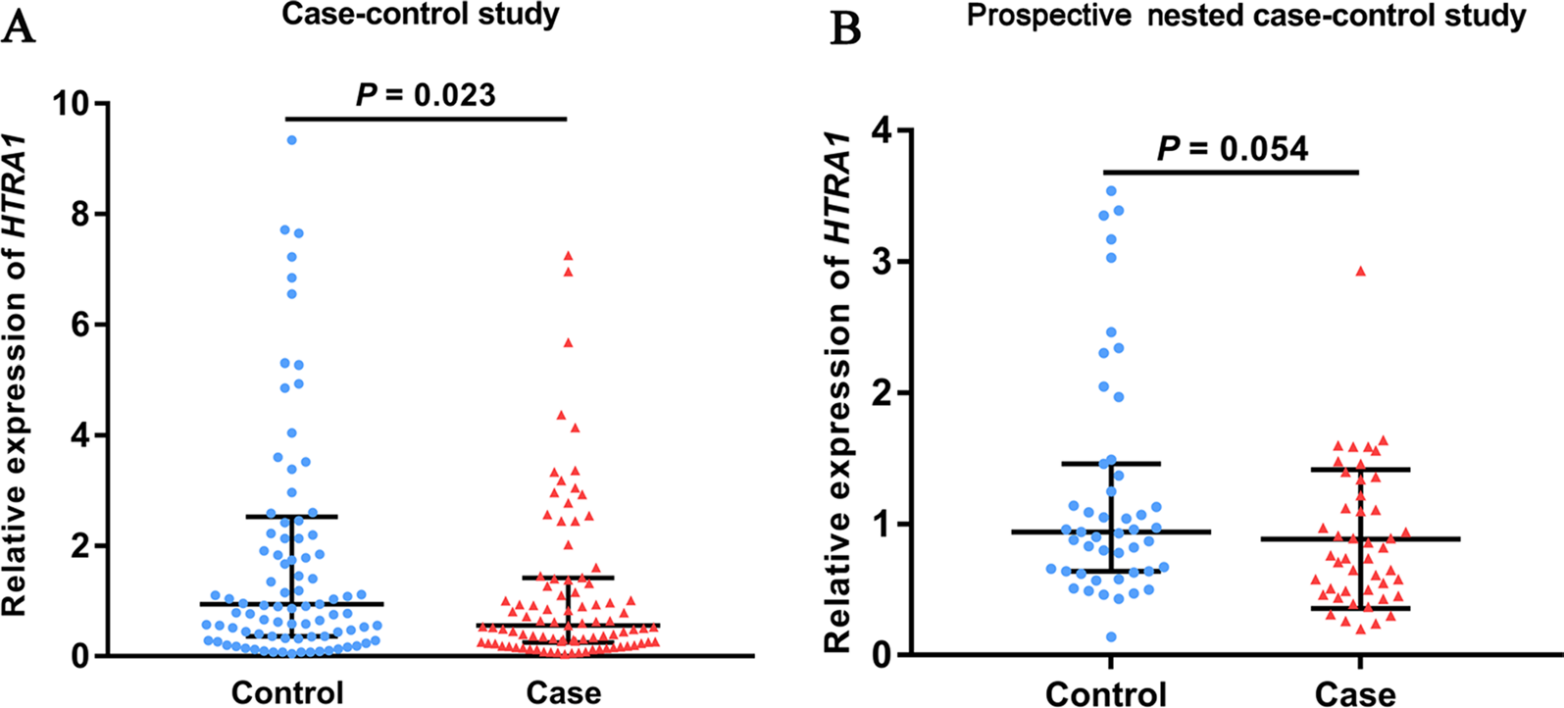
The expression of *HTRA1* in the peripheral blood leukocytes in controls and stroke cases. **A** The relative expression of *HTRA1* between stroke cases and controls in the case–control study. **B** The relative expression of *HTRA1* between stroke cases and controls in the prospective nested case–control study. The dots represent the relative expression levels of *HTRA1* in each individual. The upper line and lower line represent upper quartile and lower quartile, and the medium line represents the median. The *P* values were calculated by Mann–Whitney U tests

To understand the correlation between the methylation and expression of *HTRA1* gene, RNA was isolated from peripheral blood leukocytes of 96 stroke cases and 96 controls in the case–control study. The median of relative expression levels of *HTRA1* in the leukocytes of stroke cases was 1.68-fold lower than in the controls (*HTRA1* relative expression, median and IQR of cases: 0.56 (0.25–1.42), median and IQR of controls: 0.94 (0.36–2.52), *P* = 0.023, Fig. 2A).

Next, we plan to measure the expression of *HTRA1* in peripheral blood leukocytes in a prospective study. However, no RNA materials are available from the nested case–control study mentioned above as well as in the materials and methods. Thus, we analyzed blood RNA from 48 stroke cases who developed stroke within 1 year after the enrollment and 48 matched stroke-free controls from another prospective study in Jurong (Additional file 1: Table S7). The median of relative expression levels of *HTRA1* in the leukocytes of stroke cases was 1.24-fold lower than in the controls (*HTRA1* relative expression, median and IQR of cases: 0.76 (0.49–1.34), median and IQR of controls: 0.94 (0.64–1.46), Fig. 2B), although the *P* value did not reach significance (*P* = 0.054) probably due to the limited sample size.

### *HTRA1* methylation as a marker for the early detection of stroke

**Fig. 3:**
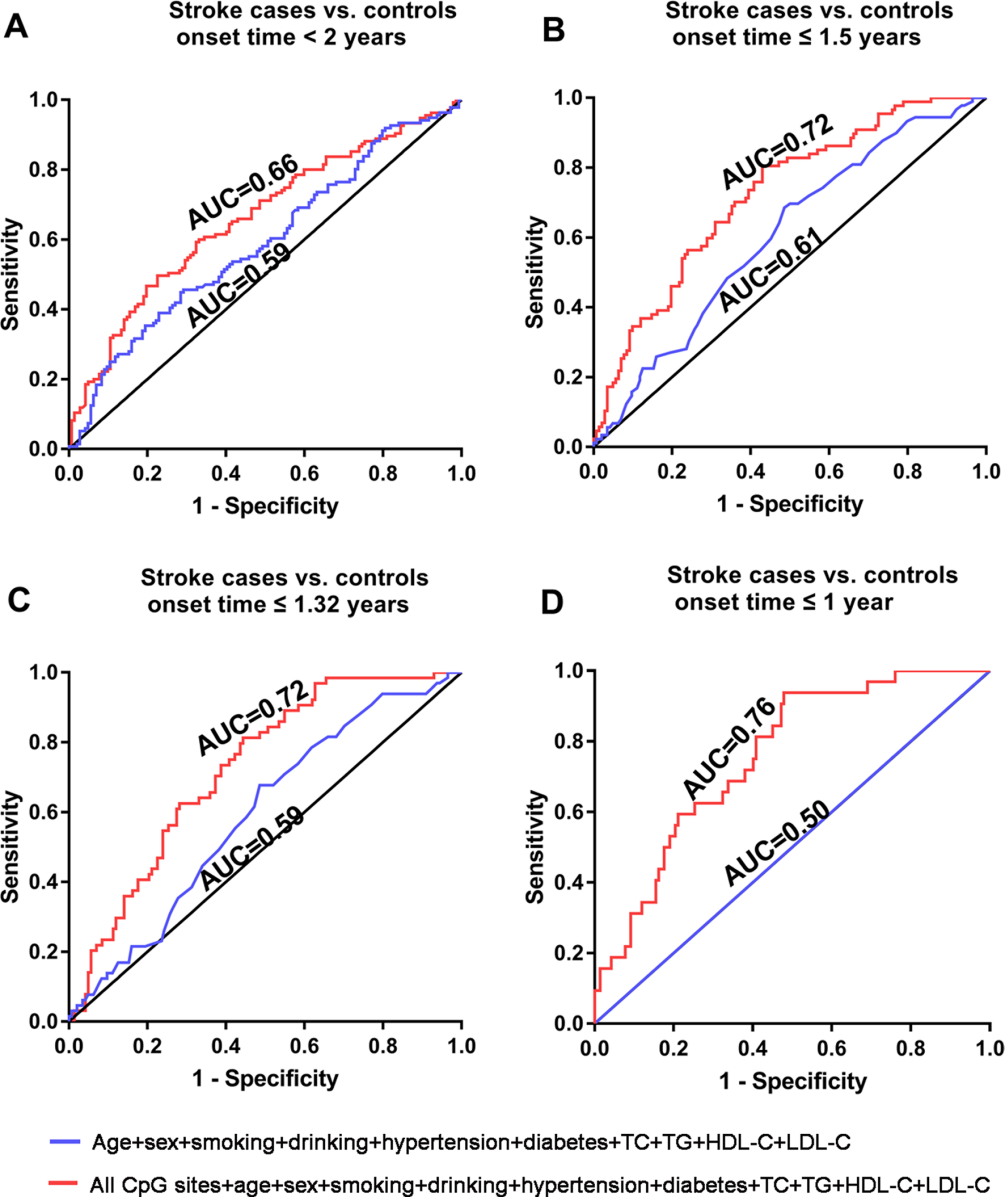
Methylation level of *HTRA1* in the peripheral blood DNA as a marker for the early detection of stroke. ROC curve analyses for the discriminatory power of *HTRA1* methylation to distinguish stroke cases with onset time **A** < 2 years, **B** ≤ 1.5 years, **C** ≤ 1.32 years, and **D** ≤ 1 year from controls. The ROC analyses were performed by logistic regression using all the analyzed CpG sites and stroke-related variables including age, sex, smoking, drinking, hypertension, diabetes, TC, TG, HDL-C and LDL-C

To estimate the potential clinical utility of *HTRA1* methylation as a marker for the early detection of stroke, ROC curve analyses were performed based on the methylation data generated from the prospective nested case–control study using all the analyzed CpG sites and stroke-related variables including age, sex, smoking, drinking, hypertension, diabetes, TC, TG, HDL-C and LDL-C by logistic regression. The combination of all the analyzed CpG sites and stroke-related variables showed better discriminatory power for differentiating stroke cases at any time of onset than stroke-related variables alone (Fig. 3A–D). For stroke cases with shorter onset time, the *HTRA1* methylation and stroke-related variables showed increasing discriminatory power for differentiating stroke cases from healthy controls (area under the curve (AUC) = 0.66, 0.72 and 0.72 for stroke cases with onset time < 2 years, ≤ 1.5 years and ≤ 1.32 years, respectively, Fig. 3A–C). Notably, the combination of *HTRA1* methylation and stroke-related variables had robust power to distinguish stroke cases with onset time ≤ 1 year from controls (AUC = 0.76, 95% CI 0.67–0.84, Fig. 3D).

## Discussion

In our study, the stroke-related altered *HTRA1* methylation in blood cells was observed in both hospital-based case–control study and prospective nested case–control study. The prospective data further disclosed that the changed *HTRA1* methylation was detectable 2 years preclinical of stroke and became more pronounced 1.5 years and 1 year before the clinical determination of stroke. These findings provided pronounced evidence that differential DNA methylation signatures in the peripheral blood could be a potential biomarker for the risk evaluation and preclinical detection of stroke.

HTRA1, a primarily secreted serine protease, degrades a number of substrates that involve in a variety of disease processes such as neurodegeneration [26], age-related macular degeneration [27], and arthritis [28]. The loss of *HTRA1* function has been reported to cause cerebral autosomal recessive arteriopathy with subcortical infarcts and leukoencephalopathy (CARASIL), an inherited form of cerebral SVD characterized by an early-onset stroke in young adults [29]. To date, little is yet known about the epigenetic impact of *HTRA1* on the initiation and progression of vascular disorders including stroke. This study was novel in reporting a significant association between aberrant blood-based *HTRA1* and increased risk of stroke, and this association was more pronounced when the baseline was closer to the onset time of stroke. The prospective results suggested that the methylation level of *HTRA1* in blood DNA might be a preclinical marker for stroke. Moreover, significantly decreased expression of *HTRA1* was observed in the stroke cases, as well as in the preclinical stroke cases, indicating that the increased *HTRA1* methylation in blood cells might be biologically meaningful. Future prospective multicenter studies are warranted to verify these findings.

Many studies disclosed that the DNA methylation in human peripheral leukocytes could vary with age [30, 31]. In our case–control study and prospective study, a significant association between methylation of *HTRA1* and stroke was mainly observed in the older population, which was in accordance with the fact that the elderly men have a higher risk of stroke [32], and further indicated that *HTRA1* methylation might be an age-dependent factor for the risk of stroke. Alternatively, the methylation pattern might be affected by lifestyle such as alcohol drinking [33] and other stroke-related factors like hypertension [34]. In our prospective nested case–control study, we observed that the methylation level of HTRA1_A_CpG_2 was positively correlated with current drinking status, while the methylation levels of CpG_3, CpG_4 and CpG_5 in the HTRA1_A amplicon were inversely correlated with hypertension. The specific mechanisms of DNA methylation and alcohol drinking as well as hypertension remained unclear. Further studies with larger sample sizes are needed to investigate the complex interactions between lifestyle, environmental factors, and DNA methylation throughout the progression of stroke.

Despite the prevalence of stroke, risk prediction and early detection remain challenging. Early detection of stroke is vital for providing timely treatment through thrombolysis, improving clinical outcomes and reducing resulting complications, and leading to better survival results and less disease burden [35, 36]. At present, magnetic resonance imaging (MRI) and computed tomography (CT) provide efficient tools for stroke diagnosis, but are only efficient at the onset of stroke. In this study, the blood-based *HTRA1* methylation can objectively predict stroke a year in advance with an area under the curve of 0.76. We should admit that the *HTRA1* methylation alone is not sufficient as a screening tool, the combination of multiple biomarkers including blood-based DNA methylation profiles and others as well as stroke-related environmental factors might be a solution for the screening and prediction of stroke in preclinic.

The long-term follow-up of stroke incidence and prospectively collected samples with careful uniform processing and storage in this study could exclude the possibility of observed differences between cases and controls owning to potential treatment or processing effects. Although with a 2-year follow-up in a cohort of 11,151 subjects, only 139 stroke cases were identified after the enrollment. This observed *HTRA1* methylation-related risk of stroke was still based on a relatively small sample size. The association between *HTRA1* methylation and stroke in the case–control study was not completely consistent with the prospective nested case–control study when each CpG was considered. This may also be attributed to the limited sample size. Further prospective studies with larger sample sizes are needed for validation and may also provide additional insights.

## Conclusions

In conclusion, this study revealed a significant correlation between altered *HTRA1* methylation and stroke in preclinic, thus suggesting the potential of blood-based DNA methylation for the risk evaluation and even prevention of stroke. Further validation studies are warranted to ensure the generalizability of our findings and evaluate the potential usage of *HTRA1* methylation as a clinical biomarker for stroke.

## Methods

### Study population

#### Hospital-based case–control study

The case–control study included 190 stroke patients and 190 controls matched for age and sex. The patients (mean age of 64.99 ± 9.85 years) were recruited from the Affiliated Hospital of Xuzhou Medical University, Affiliated Jiangning Hospital of Nanjing Medical University and Jurong People's Hospital from January 2019 to August 2020. The clinical diagnosis of stroke was based on the clinical examination and confirmed by MRI or CT. The diagnostic information and clinical examination data were obtained from hospital medical records. The demographic information including age, sex, nationality, status of smoking and status of drinking and medical history of patients were collected using standardized questionnaires. The controls (mean age of 65.89 ± 10.12 years) were recruited from a cohort study conducted from November 2018 to January 2019 in Jurong City, Jiangsu Province, and those who remained stroke-free during the 2-year follow-up were selected. Blood samples were collected after 8 h from the last meal or after an overnight fast to measure the levels of fasting plasma glucose, TC, TG, HDL-C, and LDL-C. The individuals were considered as diabetes cases in the presence of fasting plasma glucose ≥ 7.0 mmol/L or a self-reported diabetes history according to the diagnostic criteria for diabetes suggested by the American Diabetes Association. Additionally, blood samples of all the cases and controls were processed following the same protocol and stored at − 80 °C after drawing.

#### Prospective nested case–control study

Subjects for this nested case–control study were selected from an ongoing population-based cohort study conducted in Jurong City, Jiangsu Province, which has been described in our previous study [16]. Briefly, a total of 11,151 subjects aged 18 years or older were recruited in this cohort from October to November of 2015, and all the subjects were reported to be stroke-free at baseline. Detailed information on demography and status of smoking and status of drinking as well as family history were collected via questionnaires, along with the collection of blood samples and anthropometric measurements at enrollment. The incidence of stroke was identified by records from the local hospitals and health service centers in the community. All the 139 subjects who developed a stroke within 2 years after the enrollment were included in this study as cases (mean age of 67.64 ± 9.51 years), and 150 participants who remained stroke-free during the 2.71-year follow-up time were selected as controls. After excluding 6 low-quality clotted blood samples, 144 controls (mean age of 67.77 ± 9.11 years) who well matched the stroke patients by age and sex were included.

### DNA processing and bisulfite conversion

The Genomic DNA Extraction Kit (Rebece, Nanjing, China) was used to extract human peripheral blood cells’ genomic DNA as described previously [37]. Then the extracted DNA was further bisulfate-converted by EZ-96 DNA Methylation Gold Kit (Zymo Research, Orange County, USA) according to the manufacturer’s protocol. After bisulfite treatment, all non-methylated cytosine (C) bases were converted to uracil (U), whereas all methylated C bases were kept unchanged.

### MALDI-TOF mass spectrometry

DNA methylation of *HTRA1* in the peripheral blood was quantitatively measured using MALDI-TOF mass spectrometry (Agena Bioscience, San Diego, California, USA) as described by Yang et al*.* [38, 39]. Briefly, the bisulfite-treated genomic DNA was amplified by bisulfite-specific primers. The primers and sequences of polymerase chain reaction (PCR) amplicons (HTRA1-A and HTRA1-B) are presented in Additional file 1: Fig. S1. The HTRA1-A amplicon in the gene promoter region is located at 664 bp upstream of the nearby CpG island. The HTRA1-B amplicon in the first intron is located at 120 bp downstream of the nearby CpG island. Alternatively, these two amplicons are located at the so-called CpG island shores where differential methylation occurs frequently [40, 41]. There are no SNPs located at the primer regions or overlapped with any of the CpG sites in the amplicon. The PCR products were processed following shrimp alkaline phosphatase cleanup, T cleavage and Resin Clean steps. The final products were transferred to a SpectroCHIP G384 by a Nanodispenser RS1000 apparatus (Agena, USA). The chips were detected by MassARRAY spectrometry. The quantitative methylation levels of each CpG site or aggregate of multiple CpG sites were collected by EpiTYPER v1.2 software. For each batch of MassARRAY analyses, stroke case-and-control pairs were randomly arranged, and the samples were treated and analyzed in parallel in all the processes.

### Quantitative real-time PCR

Total RNA was extracted from peripheral blood leucocytes using the QIAamp RNA Blood Mini Kit (Qiagen, Catalog no. 52304, Germany) and then reverse-transcribed into cDNA using the PrimeScript™ RT Reagent Kit (Takara, RR047A, Japan). Quantitative real-time PCR was performed to determine the expressions of *HTRA1* gene and the housekeeping gene glyceraldehyde-3-phosphate dehydrogenase (*GAPDH)* as an endogenous control using 2 × SYBR Green qPCR Master Mix (Bimake, B21202, USA). The relative expression of *HTRA1* for each sample was normalized by *GAPDH* according to the 2^−ΔΔ*ct*^ method.

### Statistical analysis

All statistical analyses were conducted in SPSS version 25.0 (SPSS Inc., Chicago, USA). The methylation levels of each CpG site and gene expression data with non-Gaussian distribution were expressed as median (interquartile range). Differences between cases and controls were analyzed with Mann–Whitney U tests. The association between the level of *HTRA1* methylation and stroke was assessed using logistic regression models with covariates-adjusted odds ratios (ORs) and 95% confidence intervals (CIs). The correlation between *HTRA1* methylation and stroke-related characteristics was evaluated by Spearman’s rank correlation coefficients. Receiver operating characteristic (ROC) curve analysis was performed to assess the discriminatory power of methylation levels. A two‐tailed *P* < 0.05 was considered statistically significant.

## Supplementary Information

**Additional file 1.** **Table S1.** Methylation difference of HTRA1 between 144 controls and 139 stroke cases with onset time < 2 years in the prospective nested casecontrol study. **Table S2.** Methylation difference of *HTRA1* between 144 controls and 91 stroke cases with onset time ≤ 1.5 years in the prospective nested case-control study. **Table S3.** Methylation difference of *HTRA1* between 144 controls and 67 stroke cases with onset time ≤ 1.32 years in the prospective nested case-control study. **Table S4.** Methylation difference of *HTRA1* between 144 controls and 35 stroke cases with onset time ≤ 1 year in the prospective nested case-control study. **Table S5.** Correlation between *HTRA1* methylation and stroke-related characteristics in the case-control study. **Table S6.** Correlation between *HTRA1* methylation and stroke-related characteristics in the prospective nested case-control study. **Table S7.** Description of the subjects for RNA analysis. **Fig. S1** Amplicon and primer design for MassARRAY methylation analysis.

## Data Availability

The datasets reported in the current study are available from the corresponding author upon reasonable request.

## Abbreviations

AUC: Area under the curve
CARASIL: Cerebral autosomal recessive arteriopathy with subcortical infarcts and leukoencephalopathy
CIs: Confidence intervals
CpG: Cytosine–phosphate–guanine
CT: Computed tomography
DBP: Diastolic blood pressure
HDL-C: High-density lipoprotein cholesterol
HTRA1: HTRA serine protease 1
LDL-C: Low-density lipoprotein cholesterol
MRI: Magnetic resonance imaging
ORs: Odds ratios
PCR: Polymerase chain reaction
ROC: Receiver operating characteristic
SBP: Systolic blood pressure
SNP: Single nucleotide polymorphism
SVD: Small vessel disease
TC: Total cholesterol
TG: Triglycerides
TGF-β: Transforming growth factor-β
