# Correction : *HTRA1* methylation in peripheral blood as a potential marker for the preclinical detection of stroke: a case–control study and a prospective nested case–control study

**DOI:** 10.1186/s13148-023-01558-x

**Published:** 2023-08-29

**Authors:** Chunlan Liu, Mengxia Li, Qiming Yin, Yao Fan, Chong Shen, Rongxi Yang

**Affiliations:** 1 Department of Epidemiology and Biostatistics, School of Public Health, Nanjing Medical University, 101 Longmian Avenue, Jiangning, Nanjing, 211166 China; 2 Division of Clinical Epidemiology, Affiliated Geriatric Hospital of Nanjing Medical University, Nanjing, 211166 China

**Correction : Clinical Epigenetics (2022) 14:191** **https://doi.org/10.1186/s13148-022-01418-0**

Following publication of the original article [1], the authors have realized that ethics statement and Acknowledgement Section are incomplete. The corrected ethics statement should read as follows:

The prospective nested case–control study was approved by the Ethics Committee of Nanjing Medical University (No. 2015077). For Hospital-based case–control studies were approved by the ethics committee of Affiliated Hospital of Xuzhou Medical University (No. XYFY2018-KL078), Affiliated Jiangning Hospital of Nanjing Medical University (No.2018676), Jurong People's Hospital (No.JRSRMYY-2019-02), All the recruited participants were Chinese Han and provided written informed consent.

In full Acknowledgement statement should state:

We would like to thank Yu Liu, Min Liu and Haifeng Xu for their contribution to the study. We would also thank to Dr. Deqin Geng and Dr. Lihua Liu for their contribution in allowing blood samples and related information collection. We would also like to express our gratitude to all of the participants of the contributing studies to each of these datasets.
