# Supplementary material for: HTRA1 methylation in peripheral blood as a potential marker for the preclinical detection of stroke: a case–control study and a prospective nested case–control study
Source: Clin Epigenetics. 2022 Dec 29;14:191. doi: 10.1186/s13148-022-01418-0 (PMC9801609; doi:10.1186/s13148-022-01418-0)
Supplement: Supplementary file 1 — Additional file 1. Table S1. Methylation difference of HTRA1 between 144 controls and 139 stroke cases with onset time < 2 years in the prospective nested casecontrol study. Table S2. Methylation difference of HTRA1 between 144 controls and 91 stroke cases with onset time ≤ 1.5 years in the prospective nested case-control study. Table S3. Methylation difference of HTRA1 between 144 controls and 67 stroke cases with onset time ≤ 1.32 years in the prospective nested case-control study. Table S4. Methylation difference of HTRA1 between 144 controls and 35 stroke cases with onset time ≤ 1 year in the prospective nested case-control study. Table S5. Correlation between HTRA1 methylation and stroke-related characteristics in the case-control study. Table S6. Correlation between HTRA1 methylation and stroke-related characteristics in the prospective nested case-control study. Table S7. Description of the subjects for RNA analysis. Fig. S1 Amplicon and primer design for MassARRAY methylation analysis. [file 13148_2022_1418_MOESM1_ESM.docx]

Additional file 1: Table S1. Methylation difference of *HTRA1* between 144 controls and 139 stroke cases with onset time < 2 years in the prospective nested case-control study

| CpG sites | Controls | Stroke cases | Crude OR (95% CI) | *P - value* | OR (95% CI)^☆^ | *P - value*^☆^ | OR (95% CI)* | *P - value** |
| --- | --- | --- | --- | --- | --- | --- | --- | --- |
|  | median (IQR) | median (IQR) | per +10% methylation |  | per +10% methylation |  | per +10% methylation |  |
| HTRA1_A_CpG_1 | 0.07 (0.05-0.09) | 0.07 (0.05-0.09) | 0.73 (0.39-1.38) | 0.328 | 0.72 (0.38-1.37） | 0.321 | 0.73 (0.38-1.41) | 0.352 |
| HTRA1_A_CpG_2 | 0.77 (0.72-0.82) | 0.77 (0.72-0.82) | 0.98 (0.82-1.17) | 0.833 | 0.98 (0.82-1.17) | 0.822 | 1.01 (0.84-­1.21) | 0.935 |
| HTRA1_A_CpG_3 | 0.44 (0.41-0.49) | 0.46 (0.42-0.52) | 1.21 (0.93-1.57) | 0.151 | 1.21 (0.93-1.57) | 0.148 | 1.17 (0.90­-1.52) | 0.254 |
| HTRA1_A_CpG_4 | 0.19 (0.16-0.21) | 0.19 (0.17-0.22) | 1.18 (0.70-2.00) | 0.541 | 1.18 (0.70-2.00) | 0.542 | 1.12 (0.66-­1.91) | 0.678 |
| HTRA1_A_CpG_5 | 0.42 (0.36-0.50) | 0.40 (0.35-0.45) | 0.81 (0.65-1.00) | 0.053 | 0.80 (0.65-1.00) | 0.053 | 0.81 (0.65­-1.01) | 0.062 |
| HTRA1_A_CpG_6 | 0.09 (0.08-0.11) | 0.08 (0.07-0.10) | 0.69 (0.39-1.20) | 0.186 | 0.68 (0.39-1.19) | 0.177 | 0.64 (0.36-­1.12) | 0.119 |
| HTRA1_A_CpG_7 | 0.39 (0.34-0.43) | 0.40 (0.35-0.45) | 1.16 (0.86-1.56) | 0.323 | 1.16 (0.87-1.56) | 0.316 | 1.16 (0.86-­1.56) | 0.343 |
| HTRA1_B_CpG_1 | 0.18 (0.11-0.28) | 0.18 (0.11-0.29) | 1.10 (0.94-1.28) | 0.231 | 1.10(0.94-1.28） | 0.225 | 1.09 (0.93­-1.28) | 0.269 |
| HTRA1_B_CpG_2 | 0.12 (0.09-0.16) | 0.12 (0.08-0.17) | 1.06 (0.71-1.57) | 0.776 | 1.06 (0.71-1.58） | 0.771 | 1.10 (0.74­-1.65) | 0.634 |
| HTRA1_B_CpG_3.4 | 0.31 (0.26-0.35) | 0.32 (0.27-0.37) | 1.37 (1.00-1.87) | 0.052 | 1.37 (1.00-1.87） | 0.053 | 1.42 (1.03-­1.95) | **0.034** |
| HTRA1_B_CpG_5 | 0.34 (0.27-0.40) | 0.35 (0.27-0.44) | 1.21 (0.97-1.51) | 0.096 | 1.21 (0.97-1.52） | 0.097 | 1.22 (0.97-­1.53) | 0.088 |
| HTRA1_B_CpG_7 | 0.48 (0.40-0.53) | 0.47 (0.39-0.57) | 1.06 (0.89-1.28) | 0.511 | 1.06 (0.88-1.28） | 0.522 | 1.08 (0.90-­1.30) | 0.423 |
| HTRA1_B_CpG_8.9 | 0.31 (0.26-0.35) | 0.32 (0.27-0.37) | 1.37 (1.00-1.87) | 0.052 | 1.37 (1.00-1.87） | 0.053 | 1.42 (1.03­-1.95) | **0.034** |
| HTRA1_B_CpG_10 | 0.63 (0.54-0.69) | 0.66 (0.55-0.75) | 1.16 (0.96-1.39) | 0.126 | 1.16 (0.96-1.39） | 0.126 | 1.15 (0.95-­1.38) | 0.152 |
| HTRA1_B_CpG_11.12 | 0.75 (0.68-0.79) | 0.76 (0.69-0.82) | 1.28 (0.99-1.65) | 0.057 | 1.28 (0.99-1.65） | 0.058 | 1.25 (0.97­-1.63) | 0.089 |
| HTRA1_B_CpG_13.14.15 | 0.53 (0.46-0.61) | 0.57 (0.49-0.63) | 1.33 (1.06-1.68) | 0.014 | 1.33 (1.06-1.68） | 0.015 | 1.33 (1.06-­1.68) | **0.016** |
| HTRA1_B_CpG_16 | 0.48 (0.40-0.57) | 0.49 (0.37-0.60) | 0.96 (0.82-1.11) | 0.554 | 0.95 (0.82-1.11） | 0.539 | 0.96 (0.82­-1.12) | 0.572 |

^☆^logistic regression, adjusted for age and gender;

*logistic regression, adjusted for age, gender, smoking, drinking, hypertension, diabetes, TC, TG, HDL-C and LDL-C;

Bold values indicated *P* < 0.05Additional file 1: Table S2. Methylation difference of *HTRA1* between 144 controls and 91 stroke cases with onset time ≤ 1.5 years in the prospective nested case-control study

| CpG sites | Controls | Stroke cases | Crude OR (95% CI) | *P - value* | OR (95% CI)^☆^ | *P - value*^☆^ | OR (95% CI)* | *P - value** |
| --- | --- | --- | --- | --- | --- | --- | --- | --- |
|  | median (IQR) | median (IQR) | per +10% methylation |  | per +10% methylation |  | per +10% methylation |  |
| HTRA1_A_CpG_1 | 0.07 (0.05-0.09) | 0.07 (0.05-0.09) | 0.69 (0.33-1.42) | 0.311 | 0.68 (0.33-1.41) | 0.294 | 0.67 (0.32­-1.42) | 0.300 |
| HTRA1_A_CpG_2 | 0.77 (0.72-0.82) | 0.77 (0.72-0.82) | 0.96 (0.79-1.16) | 0.648 | 0.95 (0.79-1.16) | 0.633 | 0.97 (0.80­-1.19) | 0.782 |
| HTRA1_A_CpG_3 | 0.44 (0.41-0.49) | 0.46 (0.43-0.51) | 1.18 (0.88-1.58) | 0.282 | 1.18 (0.88-1.58) | 0.285 | 1.14 (0.84-­1.54) | 0.407 |
| HTRA1_A_CpG_4 | 0.19 (0.16-0.21) | 0.19 (0.18-0.22) | 1.23 (0.64-2.36) | 0.533 | 1.23 (0.64-2.36) | 0.535 | 1.18 (0.61­-2.30) | 0.625 |
| HTRA1_A_CpG_5 | 0.42 (0.36-0.50) | 0.40 (0.35-0.46) | 0.86 (0.68-1.09) | 0.199 | 0.86 (0.68-1.09) | 0.201 | 0.86 (0.68­-1.10) | 0.227 |
| HTRA1_A_CpG_6 | 0.09 (0.08-0.11) | 0.08 (0.07-0.09) | 0.42 (0.18-0.96) | 0.040 | 0.42 (0.18-0.96) | 0.040 | 0.39 (0.17-­0.90) | **0.027** |
| HTRA1_A_CpG_7 | 0.39 (0.34-0.43) | 0.40 (0.36-0.46) | 1.40 (1.00-1.96) | 0.050 | 1.41 (1.00-1.97) | 0.048 | 1.42 (1.01-2.01) | **0.046** |
| HTRA1_B_CpG_1 | 0.18 (0.11-0.28) | 0.17 (0.11-0.29) | 1.11 (0.94-1.30) | 0.210 | 1.11 (0.95-1.31) | 0.199 | 1.10 (0.94-­1.30) | 0.244 |
| HTRA1_B_CpG_2 | 0.12 (0.09-0.16) | 0.13 (0.08-0.17) | 1.13 (0.73-1.76) | 0.583 | 1.14 (0.73-1.77) | 0.570 | 1.20 (0.76­-1.89) | 0.433 |
| HTRA1_B_CpG_3.4 | 0.31 (0.26-0.35) | 0.33 (0.28-0.38) | 1.45 (1.02-2.06) | 0.037 | 1.45 (1.02-2.06) | 0.038 | 1.58 (1.09­-2.28) | **0.016** |
| HTRA1_B_CpG_5 | 0.34 (0.27-0.40) | 0.36 (0.26-0.46) | 1.22 (0.95-1.55) | 0.117 | 1.22 (0.95-1.56) | 0.115 | 1.23 (0.96­-1.59) | 0.103 |
| HTRA1_B_CpG_7 | 0.48 (0.40-0.53) | 0.47 (0.39-0.59) | 1.09 (0.88-1.34) | 0.435 | 1.08 (0.88-1.34) | 0.449 | 1.10 (0.89-­1.37) | 0.374 |
| HTRA1_B_CpG_8.9 | 0.31 (0.26-0.35) | 0.33 (0.28-0.38) | 1.45 (1.02-2.06) | 0.037 | 1.45 (1.02-2.06) | 0.038 | 1.58 (1.09­-2.28) | **0.016** |
| HTRA1_B_CpG_10 | 0.63 (0.54-0.69) | 0.67 (0.55-0.76) | 1.21 (0.98-1.50) | 0.084 | 1.21 (0.97-1.50) | 0.086 | 1.20 (0.96-1.49) | 0.111 |
| HTRA1_B_CpG_11.12 | 0.75 (0.68-0.79) | 0.76 (0.69-0.83) | 1.44 (1.06-1.95) | 0.020 | 1.43 (1.06-1.95) | 0.021 | 1.41 (1.03­-1.93) | **0.03** |
| HTRA1_B_CpG_13.14.15 | 0.53 (0.46-0.61) | 0.57 (0.47-0.64) | 1.41 (1.08-1.85) | 0.011 | 1.41 (1.08-1.85) | 0.012 | 1.43 (1.08­-1.88) | **0.011** |
| HTRA1_B_CpG_16 | 0.48 (0.40-0.57) | 0.49 (0.37-0.60) | 0.99 (0.84-1.17) | 0.909 | 0.99 (0.83-1.17) | 0.897 | 0.99 (0.83­-1.18) | 0.894 |

^☆^logistic regression, adjusted for age and gender;

*logistic regression, adjusted for age, gender, smoking, drinking, hypertension, diabetes, TC, TG, HDL-C and LDL-C;

Bold values indicated *P* < 0.05

Additional file 1: Table S3. Methylation difference of *HTRA1* between 144 controls and 67 stroke cases with onset time ≤ 1.32 years in the prospective nested case-control study

| CpG sites | Controls | Stroke cases | Crude OR (95% CI) | *P - value* | OR (95% CI)^☆^ | *P - value*^☆^ | OR (95% CI)* | *P - value** |
| --- | --- | --- | --- | --- | --- | --- | --- | --- |
|  | median (IQR) | median (IQR) | per +10% methylation |  | per +10% methylation |  | per +10% methylation |  |
| HTRA1_A_CpG_1 | 0.07 (0.05-0.09) | 0.07 (0.06-0.09) | 0.75 (0.34-1.66) | 0.474 | 0.74 (0.33-1.65) | 0.467 | 0.74 (0.33­-1.67) | 0.469 |
| HTRA1_A_CpG_2 | 0.77 (0.72-0.82) | 0.77 (0.73-0.81) | 0.99 (0.80-1.23) | 0.925 | 0.99 (0.8-1.23) | 0.931 | 1.01 (0.81­-1.26) | 0.930 |
| HTRA1_A_CpG_3 | 0.44 (0.41-0.49) | 0.46 (0.43-0.49) | 1.20 (0.85-1.70) | 0.307 | 1.20 (0.84-1.70) | 0.311 | 1.12 (0.78­-1.61) | 0.531 |
| HTRA1_A_CpG_4 | 0.19 (0.16-0.21) | 0.19 (0.18-0.22) | 1.18 (0.53-2.61) | 0.693 | 1.18 (0.53-2.62) | 0.690 | 1.12 (0.49­-2.55) | 0.793 |
| HTRA1_A_CpG_5 | 0.42 (0.36-0.50) | 0.39 (0.35-0.48) | 0.90 (0.70-1.16) | 0.428 | 0.90 (0.70-1.16) | 0.428 | 0.90 (0.69-­1.17) | 0.434 |
| HTRA1_A_CpG_6 | 0.09 (0.08-0.11) | 0.08 (0.07-0.09) | 0.11 (0.03-0.45) | 0.002 | 0.11 (0.03-0.45) | 0.002 | 0.10 (0.03-­0.42) | **0.002** |
| HTRA1_A_CpG_7 | 0.39 (0.34-0.43) | 0.39 (0.36-0.44) | 1.09 (0.74-1.62) | 0.655 | 1.09 (0.74-1.62) | 0.658 | 1.11 (0.74­-1.67) | 0.604 |
| HTRA1_B_CpG_1 | 0.18 (0.11-0.28) | 0.17 (0.11-0.25) | 1.09 (0.92-1.29) | 0.344 | 1.09 (0.92-1.30) | 0.336 | 1.08 (0.90­-1.29) | 0.408 |
| HTRA1_B_CpG_2 | 0.12 (0.09-0.16) | 0.14 (0.08-0.17) | 1.08 (0.65-1.79) | 0.765 | 1.08 (0.65-1.80) | 0.760 | 1.13 (0.67­-1.91) | 0.640 |
| HTRA1_B_CpG_3.4 | 0.31 (0.26-0.35) | 0.33 (0.27-0.40) | 1.47 (1.00-2.15) | 0.048 | 1.48 (1.01-2.18) | 0.045 | 1.59 (1.06-­2.38) | **0.025** |
| HTRA1_B_CpG_5 | 0.34 (0.27-0.40) | 0.36 (0.26-0.47) | 1.20 (0.92-1.57) | 0.190 | 1.21 (0.92-1.58) | 0.176 | 1.22 (0.92-­1.61) | 0.173 |
| HTRA1_B_CpG_7 | 0.48 (0.40-0.53) | 0.49 (0.40-0.61) | 1.19 (0.94-1.52) | 0.152 | 1.19 (0.94-1.52) | 0.155 | 1.21 (0.94-1.55) | 0.139 |
| HTRA1_B_CpG_8.9 | 0.31 (0.26-0.35) | 0.33 (0.27-0.40) | 1.47 (1.00-2.15) | 0.048 | 1.48 (1.01-2.18) | 0.045 | 1.59 (1.06-­2.38) | **0.025** |
| HTRA1_B_CpG_10 | 0.63 (0.54-0.69) | 0.67 (0.54-0.76) | 1.17 (0.92-1.49) | 0.190 | 1.17 (0.92-1.49) | 0.190 | 1.16 (0.90-­1.48) | 0.251 |
| HTRA1_B_CpG_11.12 | 0.75 (0.68-0.79) | 0.76 (0.65-0.83) | 1.34 (0.96-1.87) | 0.081 | 1.35 (0.97-1.88) | 0.079 | 1.33 (0.94-­1.87) | 0.103 |
| HTRA1_B_CpG_13.14.15 | 0.53 (0.46-0.61) | 0.57 (0.47-0.64) | 1.41 (1.05-1.89) | 0.024 | 1.41 (1.05-1.90) | 0.023 | 1.43 (1.05­-1.94) | **0.024** |
| HTRA1_B_CpG_16 | 0.48 (0.40-0.57) | 0.49 (0.37-0.60) | 0.99 (0.82-1.19) | 0.895 | 0.99 (0.82-1.19) | 0.897 | 0.99 (0.82-1.19) | 0.893 |

^☆^logistic regression, adjusted for age and gender;

*logistic regression, adjusted for age, gender, smoking, drinking, hypertension, diabetes, TC, TG, HDL-C and LDL-C;

Bold values indicated *P* < 0.05

Additional file 1: Table S4. Methylation difference of *HTRA1* between 144 controls and 35 stroke cases with onset time ≤ 1 year in the prospective nested case-control study

| CpG sites | Controls | Stroke cases | Crude OR (95% CI) | *P - value* | OR (95% CI)☆ | *P - value*^☆^ | OR (95% CI)* | *P - value** |
| --- | --- | --- | --- | --- | --- | --- | --- | --- |
|  | median (IQR) | median (IQR) | per +10% methylation |  | per +10% methylation |  | per +10% methylation |  |
| HTRA1_A_CpG_1 | 0.07 (0.05-0.09) | 0.07 (0.06-0.08) | 0.51 (0.17-1.53) | 0.228 | 0.52 (0.17-1.56) | 0.242 | 0.51 (0.17­-1.54) | 0.235 |
| HTRA1_A_CpG_2 | 0.77 (0.72-0.82) | 0.77 (0.74-0.81) | 1.22 (0.86-1.75) | 0.271 | 1.24 (0.86-1.77) | 0.248 | 1.24 (0.86­-1.79) | 0.250 |
| HTRA1_A_CpG_3 | 0.44 (0.41-0.49) | 0.46 (0.44-0.48) | 1.25 (0.83-1.89) | 0.291 | 1.28 (0.84-1.94) | 0.249 | 1.23 (0.80­-1.89) | 0.346 |
| HTRA1_A_CpG_4 | 0.19 (0.16-0.21) | 0.19 (0.18-0.21) | 0.74 (0.26-2.06) | 0.558 | 0.72 (0.25-2.03) | 0.530 | 0.73 (0.25­-2.09) | 0.552 |
| HTRA1_A_CpG_5 | 0.42 (0.36-0.50) | 0.39 (0.35-0.42) | 0.54 (0.35-0.83) | 0.005 | 0.53 (0.34-0.83) | 0.005 | 0.54 (0.34­-0.84) | **0.007** |
| HTRA1_A_CpG_6 | 0.09 (0.08-0.11) | 0.08 (0.08-0.09) | 0.13 (0.02-0.78) | 0.025 | 0.13 (0.02-0.76) | 0.024 | 0.12 (0.02­-0.70) | **0.018** |
| HTRA1_A_CpG_7 | 0.39 (0.34-0.43) | 0.39 (0.37-0.43) | 1.08 (0.67-1.76) | 0.745 | 1.06 (0.65-1.73) | 0.808 | 1.05 (0.63­-1.72) | 0.864 |
| HTRA1_B_CpG_1 | 0.18 (0.11-0.28) | 0.17 (0.10-0.29) | 1.13 (0.91-1.39) | 0.277 | 1.12 (0.90-1.38) | 0.304 | 1.08 (0.87­-1.36) | 0.480 |
| HTRA1_B_CpG_2 | 0.12 (0.09-0.16) | 0.12 (0.08-0.18) | 1.09 (0.58-2.06) | 0.783 | 1.08 (0.57-2.03) | 0.811 | 1.16 (0.61-­2.22) | 0.651 |
| HTRA1_B_CpG_3.4 | 0.31 (0.26-0.35) | 0.32 (0.28-0.42) | 1.55(0.96-2.50) | 0.071 | 1.64 (1.00-2.70) | 0.050 | 1.88 (1.10-­3.22) | **0.022** |
| HTRA1_B_CpG_5 | 0.34 (0.27-0.40) | 0.38 (0.29-0.47) | 1.39 (0.97-1.99) | 0.074 | 1.46 (1.00-2.12) | 0.051 | 1.50 (1.00-­2.25) | **0.048** |
| HTRA1_B_CpG_7 | 0.48 (0.40-0.53) | 0.50 (0.39-0.65) | 1.29 (0.95-1.75) | 0.109 | 1.29 (0.95-1.76) | 0.101 | 1.32 (0.95-­1.82) | 0.095 |
| HTRA1_B_CpG_8.9 | 0.31 (0.26-0.35) | 0.32 (0.28-0.42) | 1.55 (0.96-2.50) | 0.071 | 1.64 (1.00-2.70) | 0.050 | 1.88 (1.10­-3.22) | **0.022** |
| HTRA1_B_CpG_10 | 0.63 (0.54-0.69) | 0.68 (0.55-0.76) | 1.39 (0.99-1.96) | 0.060 | 1.40 (0.99-1.96) | 0.055 | 1.39 (0.98­-1.98) | 0.063 |
| HTRA1_B_CpG_11.12 | 0.75 (0.68-0.79) | 0.76 (0.70-0.83) | 1.57 (0.99-2.48) | 0.056 | 1.61 (1.01-2.55) | 0.044 | 1.60 (1.00­-2.56) | 0.052 |
| HTRA1_B_CpG_13.14.15 | 0.53 (0.46-0.61) | 0.58 (0.49-0.68) | 1.59 (1.08-2.33) | 0.018 | 1.63 (1.10-2.41) | 0.015 | 1.67 (1.11­-2.50) | **0.014** |
| HTRA1_B_CpG_16 | 0.48 (0.40-0.57) | 0.46 (0.36-0.60) | 0.97(0.76-1.24) | 0.816 | 0.98 (0.76-1.25) | 0.841 | 0.97 (0.76­-1.25) | 0.822 |

^☆^logistic regression, adjusted for age and gender;

*logistic regression, adjusted for age, gender, smoking, drinking, hypertension, diabetes, TC, TG, HDL-C and LDL-C;

Bold values indicated *P* < 0.05

Additional file 1: Table S5. Correlation between *HTRA1* methylation and stroke-related characteristics in the case-control study

| CpG sites | Current-smoking status | |  | Current-drinking status | |  | History of hypertension | |  | History of diabetes | |  | TC | |  | TG | |  | HDL-C | |  | LDL-C | |  |
| --- | --- | --- | --- | --- | --- | --- | --- | --- | --- | --- | --- | --- | --- | --- | --- | --- | --- | --- | --- | --- | --- | --- | --- | --- |
|  | Spearman  rho | *P*  value |  | Spearman  rho | *P*  value |  | Spearman  rho | *P*  value |  | Spearman  rho | *P*  value |  | Spearman  rho | *P*  value |  | Spearman  rho | *P*  value |  | Spearman  rho | *P*  value |  | Spearman  rho | *P*  value | |
| HTRA1_A_CpG_1 | -0.063 | 0.218 |  | 0.050 | 0.333 |  | 0.030 | 0.554 |  | 0.053 | 0.304 |  | -0.001 | 0.985 |  | -0.082 | 0.117 |  | 0.056 | 0.286 |  | 0.001 | 0.990 | |
| HTRA1_A_CpG_2 | -0.077 | 0.133 |  | 0.027 | 0.597 |  | 0.048 | 0.350 |  | 0.017 | 0.746 |  | -0.049 | 0.352 |  | -0.049 | 0.352 |  | -0.034 | 0.521 |  | -0.017 | 0.749 | |
| HTRA1_A_CpG_3 | -0.073 | 0.158 |  | 0.012 | 0.809 |  | -0.024 | 0.641 |  | 0.076 | 0.142 |  | 0.067 | 0.202 |  | 0.097 | 0.063 |  | -0.001 | 0.979 |  | 0.078 | 0.137 | |
| HTRA1_A_CpG_4 | -0.004 | 0.945 |  | 0.01 | 0.843 |  | 0.129 | **0.012** |  | 0.035 | 0.502 |  | 0.034 | 0.518 |  | 0.066 | 0.206 |  | 0.015 | 0.778 |  | 0.010 | 0.844 | |
| HTRA1_A_CpG_5 | -0.066 | 0.204 |  | -0.087 | 0.093 |  | 0.012 | 0.819 |  | -0.052 | 0.311 |  | 0.037 | 0.479 |  | -0.019 | 0.714 |  | 0.090 | 0.084 |  | 0.043 | 0.410 | |
| HTRA1_A_CpG_6 | -0.039 | 0.450 |  | 0.028 | 0.583 |  | 0.072 | 0.160 |  | 0.041 | 0.422 |  | 0.052 | 0.321 |  | -0.020 | 0.701 |  | 0.063 | 0.226 |  | 0.036 | 0.498 | |
| HTRA1_A_CpG_7 | -0.068 | 0.184 |  | -0.018 | 0.725 |  | -0.026 | 0.612 |  | 0.037 | 0.473 |  | 0.053 | 0.313 |  | -0.015 | 0.771 |  | 0.085 | 0.105 |  | 0.039 | 0.455 | |
| HTRA1_B_CpG_1 | 0.091 | 0.076 |  | -0.001 | 0.981 |  | 0.094 | 0.068 |  | -0.067 | 0.196 |  | 0.000 | 0.997 |  | -0.027 | 0.600 |  | -0.009 | 0.868 |  | -0.039 | 0.455 | |
| HTRA1_B_CpG_2 | -0.042 | 0.419 |  | -0.012 | 0.810 |  | 0.068 | 0.186 |  | 0.023 | 0.655 |  | 0.090 | 0.084 |  | 0.072 | 0.167 |  | 0.139 | **0.008** |  | 0.059 | 0.257 | |
| HTRA1_B_CpG_3.4 | 0.084 | 0.101 |  | 0.036 | 0.491 |  | 0.011 | 0.838 |  | -0.006 | 0.912 |  | -0.007 | 0.901 |  | 0.078 | 0.137 |  | -0.004 | 0.940 |  | -0.044 | 0.405 | |
| HTRA1_B_CpG_5 | 0.061 | 0.234 |  | 0.017 | 0.739 |  | -0.003 | 0.959 |  | -0.032 | 0.541 |  | -0.007 | 0.891 |  | 0.089 | 0.089 |  | -0.003 | 0.955 |  | 0.011 | 0.838 | |
| HTRA1_B_CpG_7 | 0.043 | 0.410 |  | 0.034 | 0.508 |  | 0.011 | 0.827 |  | 0.036 | 0.489 |  | 0.007 | 0.890 |  | 0.053 | 0.313 |  | -0.015 | 0.772 |  | -0.002 | 0.967 | |
| HTRA1_B_CpG_8.9 | 0.084 | 0.101 |  | 0.036 | 0.491 |  | 0.011 | 0.838 |  | -0.006 | 0.912 |  | -0.007 | 0.901 |  | 0.078 | 0.137 |  | -0.004 | 0.940 |  | -0.044 | 0.405 | |
| HTRA1_B_CpG_10 | 0.085 | 0.100 |  | 0.040 | 0.440 |  | 0.015 | 0.769 |  | 0.046 | 0.371 |  | 0.030 | 0.569 |  | 0.068 | 0.196 |  | -0.005 | 0.919 |  | 0.012 | 0.819 | |
| HTRA1_B_CpG_11.12 | 0.087 | 0.091 |  | 0.003 | 0.955 |  | 0.043 | 0.406 |  | -0.031 | 0.546 |  | 0.051 | 0.335 |  | 0.091 | 0.080 |  | -0.007 | 0.893 |  | 0.019 | 0.719 | |
| HTRA1_B_CpG_13.14.15 | 0.106 | **0.040** |  | 0.046 | 0.373 |  | 0.038 | 0.463 |  | -0.007 | 0.890 |  | 0.130 | **0.013** |  | 0.116 | **0.027** |  | -0.005 | 0.917 |  | 0.079 | 0.131 | |
| HTRA1_B_CpG_16 | 0.049 | 0.345 |  | 0.030 | 0.566 |  | 0.055 | 0.283 |  | -0.028 | 0.589 |  | 0.081 | 0.123 |  | -0.052 | 0.320 |  | 0.054 | 0.301 |  | 0.078 | 0.134 | |

Bold values indicated *P* < 0.05

Additional file 1: Table S6. Correlation between *HTRA1* methylation and stroke-related characteristics in the prospective nested case-control study

| CpG sites | Current-smoking status | |  | Current-drinking status | |  | History of hypertension | |  | History of diabetes | |  | TC | |  | TG | |  | HDL-C | |  | LDL-C | |
| --- | --- | --- | --- | --- | --- | --- | --- | --- | --- | --- | --- | --- | --- | --- | --- | --- | --- | --- | --- | --- | --- | --- | --- |
|  | Spearman  rho | *P*  value |  | Spearman  rho | *P*  value |  | Spearman  rho | *P*  value |  | Spearman  rho | *P*  value |  | Spearman  rho | *P*  value |  | Spearman  rho | *P*  value |  | Spearman  rho | *P*  value |  | Spearman  rho | *P*  value |
| HTRA1_A_CpG_1 | -0.049 | 0.417 |  | -0.015 | 0.796 |  | -0.099 | 0.096 |  | 0.048 | 0.419 |  | -0.054 | 0.364 |  | 0.001 | 0.986 |  | -0.073 | 0.220 |  | -0.046 | 0.441 |
| HTRA1_A_CpG_2 | 0.087 | 0.147 |  | 0.199 | **0.001** |  | 0.075 | 0.214 |  | -0.029 | 0.626 |  | -0.061 | 0.312 |  | 0.072 | 0.229 |  | -0.067 | 0.268 |  | -0.068 | 0.255 |
| HTRA1_A_CpG_3 | 0.007 | 0.908 |  | -0.018 | 0.762 |  | -0.121 | **0.043** |  | 0.024 | 0.688 |  | 0.011 | 0.849 |  | -0.053 | 0.371 |  | -0.031 | 0.599 |  | 0.002 | 0.969 |
| HTRA1_A_CpG_4 | 0.076 | 0.201 |  | -0.006 | 0.927 |  | -0.263 | **7.00E-10** |  | -0.094 | 0.114 |  | 0.001 | 0.982 |  | -0.093 | 0.117 |  | 0.005 | 0.937 |  | 0.015 | 0.799 |
| HTRA1_A_CpG_5 | -0.014 | 0.817 |  | -0.009 | 0.882 |  | -0.198 | **0.001** |  | 0.057 | 0.336 |  | -0.026 | 0.659 |  | -0.043 | 0.468 |  | -0.084 | 0.161 |  | -0.004 | 0.942 |
| HTRA1_A_CpG_6 | 0.052 | 0.381 |  | 0.112 | 0.061 |  | 0.045 | 0.452 |  | -0.017 | 0.781 |  | -0.071 | 0.232 |  | -0.045 | 0.453 |  | 0.05 | 0.399 |  | -0.081 | 0.176 |
| HTRA1_A_CpG_7 | 0.015 | 0.802 |  | -0.004 | 0.943 |  | -0.068 | 0.257 |  | -0.017 | 0.775 |  | -0.054 | 0.366 |  | -0.116 | 0.053 |  | 0.048 | 0.422 |  | -0.055 | 0.360 |
| HTRA1_B_CpG_1 | -0.009 | 0.877 |  | 0.107 | 0.074 |  | 0.096 | 0.110 |  | 0.010 | 0.873 |  | 0.011 | 0.852 |  | 0.049 | 0.417 |  | -0.062 | 0.297 |  | 0.006 | 0.915 |
| HTRA1_B_CpG_2 | 0.007 | 0.910 |  | 0.033 | 0.580 |  | -0.055 | 0.361 |  | -0.074 | 0.215 |  | 0.001 | 0.987 |  | -0.058 | 0.328 |  | 0.014 | 0.816 |  | -0.003 | 0.959 |
| HTRA1_B_CpG_3.4 | -0.027 | 0.650 |  | 0.055 | 0.361 |  | -0.063 | 0.296 |  | -0.040 | 0.500 |  | 0.027 | 0.654 |  | -0.039 | 0.512 |  | 0.007 | 0.913 |  | 0.031 | 0.610 |
| HTRA1_B_CpG_5 | 0.100 | 0.093 |  | 0.070 | 0.240 |  | -0.009 | 0.879 |  | -0.014 | 0.811 |  | 0.028 | 0.641 |  | -0.002 | 0.978 |  | 0.007 | 0.905 |  | 0.016 | 0.791 |
| HTRA1_B_CpG_7 | 0.089 | 0.138 |  | 0.069 | 0.248 |  | -0.076 | 0.204 |  | -0.087 | 0.149 |  | 0.055 | 0.359 |  | -0.024 | 0.691 |  | 0.062 | 0.304 |  | 0.027 | 0.659 |
| HTRA1_B_CpG_8.9 | -0.027 | 0.650 |  | 0.055 | 0.361 |  | -0.063 | 0.296 |  | -0.040 | 0.500 |  | 0.027 | 0.654 |  | -0.039 | 0.512 |  | 0.007 | 0.913 |  | 0.031 | 0.610 |
| HTRA1_B_CpG_10 | 0.001 | 0.990 |  | 0.020 | 0.741 |  | -0.016 | 0.795 |  | 0.021 | 0.725 |  | 0.085 | 0.156 |  | -0.019 | 0.755 |  | 0.009 | 0.875 |  | 0.087 | 0.144 |
| HTRA1_B_CpG_11.12 | 0.011 | 0.850 |  | 0.063 | 0.291 |  | 0.019 | 0.748 |  | 0.036 | 0.547 |  | 0.084 | 0.161 |  | -0.029 | 0.623 |  | -0.003 | 0.963 |  | 0.075 | 0.207 |
| HTRA1_B_CpG_13.14.15 | 0.001 | 0.985 |  | 0.068 | 0.256 |  | 0.002 | 0.972 |  | -0.010 | 0.870 |  | 0.083 | 0.167 |  | -0.002 | 0.969 |  | 0.018 | 0.758 |  | 0.063 | 0.289 |
| HTRA1_B_CpG_16 | 0.048 | 0.420 |  | 0.100 | 0.093 |  | 0.044 | 0.461 |  | -0.057 | 0.339 |  | 0.010 | 0.866 |  | -0.045 | 0.448 |  | 0.018 | 0.758 |  | 0.008 | 0.890 |

Bold values indicated *P* < 0.05

| Additional file 1: Table S7. Description of the subjects for RNA analysis | | | | |
| --- | --- | --- | --- | --- |
| Characteristics | Controls | Stroke cases | t/χ2 | *P -*value |
|  | (n=48) | (n=48) |  |  |
| Age (year) | 67.98±8.41 | 67.46±8.50 | 0.302 | 0.764 |
| Gender |  |  |  |  |
| Male | 21 (43.8%) | 23 (47.9%) | 0.168 | 0.838 |
| Female | 27 (56.2%) | 25 (52.1%) |  |  |
| Smoking status |  |  |  |  |
| Yes | 25 (52.1%) | 28 (58.3%) | 0.515 | 0.633 |
| No | 23 (47.9%) | 20 (41.7%) |  |  |
| Drinking status |  |  |  |  |
| Yes | 35 (72.9%) | 40 (83.3%) | 1.524 | 0.324 |
| No | 13 (27.1%) | 8 (16.7%) |  |  |
| History of hypertension |  |  |  |  |
| Yes | 20 (41.7%) | 14 (29.2%) | 1.639 | 0.286 |
| No | 28 (58.3%) | 34 (70.8%) |  |  |

Additional file 1: Figure

**a**

CTCTGCAGTCTGGCTGCTCAGGGGGATGGAGGAGGATGGGGCTTGCAGGCAGGCACAGCCGGTTTGGAAGGTGTCCTGGCTCCAGCACTCAACTTGTGGCATGACACTCAGGGGAGTCACCCTTTCTGGCCCTCAGTTTTCTCCTCTGTAAAATAAGGAAATTCGACAAAAATAAGTTATCACCAATTTATTCACCACCACGTGTCCTTTTAATTCCATCTGCCCCAGGACTGCAGTGGGGAGTCTTTCTTTGCCGATGGACTTCGAAAATCATCACCCCCCGCATTTTTTGTGGTTAACCACAGCTAGCCAGGAACTCAGGGCTTCTCATCAGCAGAGGCCTCTGCCTCTCCAGTATCCAGGGCCGAATGGATTTTGGGTCAAGGCCTCCC

**b**

GGTTAGTGATGAGGAAGAAGTCTACCCCTCTGTTCCTACAGCCGCACACAGGACCTGTTCTGGCAGGGGAGACGGTGGTGATGGGGGAAGGAGTGGAATGGAGCAATGTCTAACTCTCTCGCGGGACCTTCCGGAGAGATGCTCCTCATCTTCAGGCAGAGGCCATGTGGAAAAATAATATCGAGTTCAGCAGCGGCCAGCCCCGCGTTGTAGGAACCAGACAGCGGGGCTTGGCAGTGCGCTTGGGCGCAGCCGTGCCGCTGCTGCCGGACCCCAGTGCTGCCTCCTCAACACGGGCAGTGCCAGGAGAGGGGCATAGGGGAGCACAGTGCAGAGGGACTGGT

**c**

**Bisulfite-specific primers for HTRA1-A and HTRA1-B amplicons**

| Amplicon | Primers | Sequences |
| --- | --- | --- |
| HTRA1-A | sense | 5´-aggaagagagTTTTGTAGTTTGGTTGTTTAGGGG-3´ |
|  | antisense | 5´-cagtaatacgactcactatagggagaaggctAAAAAACCTTAACCCAAAATCCAT-3´ |
| HTRA1-B | sense | 5´-aggaagagagGGTTAGTGATGAGGAAGAAGTTTATTTT-3´ |
|  | antisense | 5´-cagtaatacgactcactatagggagaaggctACCAATCCCTCTACACTATACTCCC-3´ |

**Additional file 1: Fig. S1** Amplicon and primer design for MassARRAY methylation analysis. (a) Sequences of HTRA1-A amplicon examined by MassARRAY (chr10:124219283-124219675, build 37/hg19, defined by the UCSC Genome Browser). The MassARRAY assay determined the methylation levels of seven CpGs and yielded seven distinguishable peaks. CpG sites that could be measured are in blue. (b) Sequences of HTRA1-B amplicon for MassARRAY methylation analysis (chr7:124222360-124222704, build 37/hg19, defined by the UCSC Genome Browser). The MassARRAY assay determined the methylation levels of fifteen CpGs and yielded ten distinguishable peaks. Three peaks contain two adjacent CpG sites such as,CpG_3 and CpG_4, CpG_8 and CpG_9, CpG_11 and CpG_12. One peak contains threeCpG sites: CpG_13, CpG_14 and CpG_15. The rest six peaks contains only one single CpG site. Measurable CpG sites are in green. (c) The bisulfite-specific primers for HTRA1-A and HTRA1-B amplicons. Uppercase letters indicate the sequence specific primer regions, and non-specific tags are shown in lowercase letters. There are no known SNPs located at the primer regions, or overlapped with any of the CpGs in the two amplicons.
